# Between but Not Within-Species Variation in the Distribution of Fitness Effects

**DOI:** 10.1093/molbev/msad228

**Published:** 2023-10-13

**Authors:** Jennifer James, Chedly Kastally, Katharina B Budde, Santiago C González-Martínez, Pascal Milesi, Tanja Pyhäjärvi, Martin Lascoux, GenTree Consortium, Paraskevi Alizoti, Ricardo Alía, Olivier Ambrosio, Filippos A Aravanopoulos, Georg von Arx, Albet Audrey, Francisco Auñón, Camilla Avanzi, Evangelia Avramidou, Francesca Bagnoli, Marko Bajc, Eduardo Ballesteros, Evangelos Barbas, José M García del Barrio, Cristina C Bastias, Catherine Bastien, Giorgia Beffa, Raquel Benavides, Vanina Benoit, Frédéric Bernier, Henri Bignalet, Guillaume Bodineau, Damien Bouic, Sabine Brodbeck, William Brunetto, Jurata Buchovska, Corinne Buret, Melanie Buy, Ana M Cabanillas-Saldaña, Bárbara Carvalho, Stephen Cavers, Fernando Del Caño, Sandra Cervantes, Nicolas Cheval, José M Climent, Marianne Correard, Eva Cremer, Darius Danusevičius, Benjamin Dauphin, Jean-Luc Denou, Bernard Dokhelar, Alexis Ducousso, Bruno Fady, Patricia Faivre-Rampant, Anna-Maria Farsakoglou, Patrick Fonti, Ioannis Ganopoulos, Olivier Gilg, Nicolas De Girardi, René Graf, Alan Gray, Delphine Grivet, Felix Gugerli, Christoph Hartleitner, Katrin Heer, Enja Hollenbach, Agathe Hurel, Bernard Issenhuth, Florence Jean, Véronique Jorge, Arnaud Jouineau, Jan-Philipp Kappner, Robert Kesälahti, Florian Knutzen, Sonja T Kujala, Timo A Kumpula, Katri Kärkkäinen, Mariaceleste Labriola, Celine Lalanne, Johannes Lambertz, Gregoire Le-Provost, Vincent Lejeune, Isabelle Lesur-Kupin, Joseph Levillain, Mirko Liesebach, David López-Quiroga, Ermioni Malliarou, Jérémy Marchon, Nicolas Mariotte, Antonio Mas, Silvia Matesanz, Benjamin Meier, Helge Meischner, Célia Michotey, Sandro Morganti, Tor Myking, Daniel Nievergelt, Anne Eskild Nilsen, Eduardo Notivol, Dario I Ojeda, Sanna Olsson, Lars Opgenoorth, Geir Ostreng, Birte Pakull, Annika Perry, Sara Pinosio, Andrea Piotti, Christophe Plomion, Nicolas Poinot, Mehdi Pringarbe, Luc Puzos, Annie Raffin, José A Ramírez-Valiente, Christian Rellstab, Dourthe Remi, Oliver Reutimann, Sebastian Richter, Juan J Robledo-Arnuncio, Odile Rogier, Elisabet Martínez Sancho, Outi Savolainen, Simone Scalabrin, Volker Schneck, Silvio Schueler, Ivan Scotti, Sergio San Segundo, Vladimir Semerikov, Lenka Slámová, Ilaria Spanu, Jørn Henrik Sønstebø, Jean Thevenet, Mari Mette Tollefsrud, Norbert Turion, Fernando Valladares, Giovanni G Vendramin, Marc Villar, Marjana Westergren, Johan Westin

**Affiliations:** Department of Ecology and Genetics, Uppsala University, Uppsala, Sweden; Swedish Collegium of Advanced Study, Uppsala University, Uppsala, Sweden; Department of Forest Sciences, University of Helsinki, Helsinki, Finland; Viikki Plant Science Centre, University of Helsinki, Helsinki, Finland; Department of Forest Genetics and Forest Tree Breeding, Georg-August-University Goettingen, Goettingen, Germany; Center of Biodiversity and Sustainable Land Use (CBL), University of Goettingen, Goettingen, Germany; National Research Institute for Agriculture, Food and the Environment (INRAE), University of Bordeaux, BIOGECO, Cestas, France; Department of Ecology and Genetics, Uppsala University, Uppsala, Sweden; Science for Life Laboratory (SciLifeLab), Uppsala University, Uppsala, Sweden; Department of Forest Sciences, University of Helsinki, Helsinki, Finland; Viikki Plant Science Centre, University of Helsinki, Helsinki, Finland; Department of Ecology and Genetics, Uppsala University, Uppsala, Sweden

**Keywords:** DFE, deleterious mutations, population structure, forest trees

## Abstract

New mutations provide the raw material for evolution and adaptation. The distribution of fitness effects (DFE) describes the spectrum of effects of new mutations that can occur along a genome, and is, therefore, of vital interest in evolutionary biology. Recent work has uncovered striking similarities in the DFE between closely related species, prompting us to ask whether there is variation in the DFE among populations of the same species, or among species with different degrees of divergence, that is whether there is variation in the DFE at different levels of evolution. Using exome capture data from six tree species sampled across Europe we characterized the DFE for multiple species, and for each species, multiple populations, and investigated the factors potentially influencing the DFE, such as demography, population divergence, and genetic background. We find statistical support for the presence of variation in the DFE at the species level, even among relatively closely related species. However, we find very little difference at the population level, suggesting that differences in the DFE are primarily driven by deep features of species biology, and those evolutionarily recent events, such as demographic changes and local adaptation, have little impact.

## Introduction

The distribution of fitness effects (DFE) of new mutations, that is, the proportion of new mutations that are expected to be adaptive, neutral, slightly deleterious, or strongly deleterious, is at the heart of any evolutionary model, yet, in spite of recent progress (for a review, see Johri et al. 2022) it is still hard to estimate and is poorly understood. While there is variation in the DFE across distantly related species with dissimilar biological features (Huber et al. 2017), on shorter evolutionary timescales it is not clear how the DFE might come to differ among species or populations, although we can make some predictions from the Nearly Neutral Theory (Ohta 1973). In particular, the strength of selection acting on new mutations is expected to scale with effective population size, *N_e_*, and, therefore, to be affected by demographic processes. We also expect that the fraction of mutations inferred to be nearly neutral, that is, slightly deleterious, will be related to proxies of *N_e_*. In particular, the ratio of slightly deleterious to neutral diversity will be smaller in high *N_e_* populations (Welch et al. 2008).

Despite these predictions, empirical evidence has been mixed. Major evolutionary transitions do affect the DFE. For instance, a shift in mating systems from outcrossing to selfing leads to a lower *N_e_* and a significant increase in the fraction of slightly deleterious mutations (e.g. Douglas et al. 2015), as predicted under the Nearly Neutral Theory. However, a number of studies have found that across closely related species, the DFE and related summary statistics, such as the ratio of nonsynonymous to synonymous nucleotide diversity, *π_N_*/*π_S_*, are remarkably stable (Grivet et al. 2017; Castellano et al. 2019; Liu et al. 2022), even when comparing domesticated species and their wild relatives (Chen et al. 2017). In the latter, domestication has a very strong effect on synonymous nucleotide diversity but the ratio of nonsynonymous to synonymous nucleotide variation, a good proxy of the slightly deleterious class of mutations for populations at demographic equilibrium (Ohta 1973), was barely affected. Additionally, while some studies have found associations between parameters associated with the DFE and demographic processes such as range expansion (González-Martínez et al. 2017; Willi et al. 2020), others have not (Takou et al. 2021).

These contrasting results may reflect real biological and demographic differences across species and populations. Species may also experience different environmental conditions across their ranges, which could result in changes in the parameters of the DFE. For example, Martin and Lenormand (2006) found evidence to support a scenario in which mutations have more variable fitness effects when an organism exists in an environment to which it is less well adapted. They interpreted this result in terms of a simple fitness landscape model. A recent study in *Arabidopsis thaliana* (Weng et al. 2021) also found that mutational variance was greater in populations growing in stressful environments in which their fitness was low. However, not all of the results in Weng et al. agree with the predictions of a simple fitness landscape model. For example, the authors found that beneficial mutations were more common in populations in less stressful environments. Additionally, a review of the impact of environment on the effects of new mutations found that environmental stress can both decrease and increase the mean strength of selection acting on new mutations, as well as its variance (Agrawal and Whitlock 2010). Population differentiation may also be important, with more differentiated populations appearing to have less similar strengths of selection acting on shared mutations than less differentiated populations (Huang et al. 2021). Whether this could lead to differences in the DFE between populations given enough evolutionary time has not yet been systematically investigated.

However, contrasting results across species and populations might also be due to differences in metrics used to characterize patterns of deleterious and neutral diversity. It has been argued that while summary statistics such as the ratio of nonsynonymous to synonymous nucleotide diversity provide a good measure of the efficiency of selection, they are poor measures of the deleterious genetic load experienced by a population due to the effects of demography and nonequilibrium dynamics. For instance, after a demographic event, slightly deleterious nonsynonymous mutations will reach their equilibrium frequency spectra more rapidly than synonymous mutations, simply because the equilibrium frequencies of slightly deleterious mutations are lower (Simons et al. 2014; Simons and Sella 2016). Counts of nonsynonymous derived alleles are more robust to nonequilibrium dynamics, and give a good measure of load if mutations are deleterious, and their effects are additive. Therefore, metrics such as *R_xy_*, which were specifically developed for the purpose of estimating asymmetries in counts of derived mutations between populations, provide a better proxy of genetic load (Do et al. 2015). A combination of such metrics, in addition to those based on the site frequency spectrum, may allow for a greater understanding of how new mutations affect the molecular evolution of populations and species differ.

In the present study, we investigated variation in the DFE at both the species and population levels by leveraging exome capture data collected from range-wide populations of six forest tree species, comprising four angiosperms and three conifers, at different degrees of phylogenetic distance. These trees are keystones of European forests with a range of life history traits. All species are widely distributed, but there are marked differences in levels of population differentiation within species (see supplementary table S1, Supplementary Material online for details). By using orthologous genomic regions, we were able to compare the DFE among species while controlling for gene content. Additionally, all species have been sampled broadly across their natural ranges, following the same sampling scheme, providing us with an ideal dataset to assess the constancy of the DFE at the within-species level. Finally, we also explored variation in patterns of genetic load between populations.

## Methods

### Samples

The data consists of six wind-pollinated forest tree species (6), two conifers (*Picea abies* and *Pinus pinaster*), and four angiosperms (*Betula pendula*, *Fagus sylvatica*, *Populus nigra*, and *Quercus petraea*), distributed across Eurasia from the boreal to the Mediterranean region, and with either animal-, wind-, or water-dispersed seeds. The species vary in both life history and population structure (Milesi et al. 2023; see supplementary table S1, Supplementary Material online for details).

### Sequencing and SNP Calling

Sequencing and single nucleotide polymorphism (SNP) calling were as described in Milesi et al. (2023). Briefly, the data are the result of targeted nuclear DNA sequencing (∼10,000 species-specific probes that covered ∼3 Mb of sequence) on a total of 3,407 adult trees collected from 19 to 26 locations per species (∼25 samples each) across their distribution range. The targeted regions primarily consisted orthologous regions among species, in addition to regions that had previously been identified as targets of selection. Site-based annotation (4-fold degenerate and 0-fold degenerate sites) of detected SNPs was generated using the Python script NewAnnotateRef.py available at https://github.com/fabbyrob/science/blob/master/pileup_analyzers/NewAnnotateRef.py (Williamson et al. 2014). Detected SNPs were functionally annotated in order to predict their effects on protein sequences using the tool ANNOVAR (Wang et al. 2010). SNPs were classified as “noncoding”; “coding 4-fold degenerate synonymous”; “coding 0-fold degenerate nonsynonymous”; and “nonsense” (determining a premature STOP codon or a STOP loss). Filtering steps were applied in order to remove incorrectly assigned or clear hybrid samples. Full documentation of bioinformatics pipelines used to generate these VCF files is available at https://github.com/GenTree-h2020-eu/GenTree. The VCF files used in the present study correspond to version 5.3.2, available at https://entrepot.recherche.data.gouv.fr/dataset.xhtml?persistentId=doi:10.57745/DV2X0M. In order to be included in our analyses, both polymorphic and monomorphic sites had to have a call depth >8 or genotype quality >20. Loci with >50% missing calls were also removed. SNPs and monomorphic sites were further restricted to those that are either 4-fold or 0-fold degenerate. An additional subdivision of our SNP dataset was created, which included only those SNPs that occur in orthologous genomic regions found in all six tree species.

### SNP Polarization

To increase the power of our DFE estimation methods, we inferred the ancestral state at each SNP. This was achieved by considering the state of the site in either a single outgroup species (two species in our dataset; see supplementary table S2, Supplementary Material online for details) or two outgroup species (four species; see supplementary table S2, Supplementary Material online for details). For each species, we, therefore, mapped the genome of one or more outgroup species to the same reference genome used for SNP calling for that species using the bwa software package (Li and Durbin 2009); for further details and commands used, see supplementary table S2, Supplementary Material online. We also retained SNP sites that could not be matched to a site in an outgroup species (see in the following), due, for example, to being missing in the outgroup species genome. We used the maximum likelihood method implemented in Est-SFS (Keightley and Jackson 2018) for assigning the ancestral allele at polymorphic sites, assuming the Kimura 2-parameter substitution model. To conduct this step, we first down-sampled to a maximum number of 100 haplotypes per species by sampling randomly from a hypergeometric distribution to account for missing data and to not exceed the maximum permissible number of haplotypes for Est-SFS. We then used the probability associated with the state of each SNP to assign likely ancestral states, removing SNPs for which the probability of the major allele being the ancestral state was between 0.4 and 0.6, and which we are therefore not able to polarize with confidence. So, SNPs for which there was no outgroup information available could therefore still be assigned an ancestral state based on their minor allele frequency; however, we note that this is a small fraction of SNPs, and that all downstream analyses account for errors in ancestral state identification. We used a model averaging procedure to assess the effect of accounting for error in ancestral state identification on DFE inference (see DFE Inference); additionally, we assessed how restricting our dataset to GC-conservative mutations, which are less likely to be affected by polarization error due to the exclusion of CpG hypermutable sites, affects our results.

### Grouping Samples

For downstream analyses, we were interested in investigating variation in the DFE across a species range. DFE inference power depends on the number of sequenced individuals, and number of available SNPs; we, therefore, pooled individuals into groups based on sampling location (see supplementary fig. S1, Supplementary Material online for the map of sampling locations). This was first achieved by taking all individuals per country; subsequently, if multiple distinct admixture groups were present in this “country” pool of individuals, as identified in Milesi et al. (2023), this pool was subdivided further based on the admixture groups. If any pool contained fewer than 20 individuals it was not included in our analysis in the interest of maintaining sufficient power to achieve accurate results. We will refer to these pools as “populations”, full details of which can be found in supplementary table S3, Supplementary Material online. We also calculated the mean latitude and longitude of each sampling location per population.

### Summary Statistics

We inferred a number of standard population genetic summary statistics including Wright's fixation index, *F*_ST_ (as calculated over 4-fold sites), and 0- and 4-fold pairwise nucleotide site diversity, *π*_0_ and *π*_4_, respectively, after first projecting our data down to an SFS of 40 haplotypes, that is, 20 individuals per species or population, to account for any sites with missing data. Projecting takes the average across every possible resampling of the data, as implemented in Python using functions in the δaδi package (Gutenkunst et al. 2010). Those pairwise nucleotide diversity estimates were then used to calculate the ratio *π*_0_/*π*_4_. For each population, pairwise *F*_ST_ was calculated with Python scripts, as implemented in δaδi (Gutenkunst et al. 2010). For each species, we identified the median longitude and latitude of sampling locations, and chose as a reference the pooled population sampled closest to this location, which represents a “central” population to the species range. These “central” populations were DE for *B. pendula*, CH for *F. sylvatica*, LT for *P. abies*, FR-North for *P. pinaster*, IT-North for *P. nigra*, and CH for *Q. petraea* (for location details, see supplementary table S3, Supplementary Material online for details).

Finally, we also inferred *R_xy_*, an estimator of the differences of genetic load between populations, as defined in Do et al. (2015). Briefly, *R_xy_* measures the average difference in the accumulation of mutations between two genomes sampled in different populations at all sites for which the ancestral state is known. One counts the number of derived mutations in genome *x* that are not present in genome *y* and vice versa, and *R_xy_* is defined as the ratio of these two counts. If selection has been equally effective and mutation rates have been the same since the populations diverged, *R_xy_* is expected to equal 1. This statistic was shown to be monotonically related to the difference in mutation load between the two populations. We followed Do et al. (2015) in calculating confidence intervals on this estimate using a weighted block jack-knife procedure whereby SNP data was divided into 100 “consecutive” blocks and *R_xy_* was recalculated, removing one block per run. Each VCF was first split into chunks of length 2 Mb, based on the SNP position in the assembled genome, and then these chunks were combined into 100 groups of similar length. This grouping was done such that consecutive parts of the genome were kept together, although small scaffolds meant that occasionally different scaffolds were combined into a single group. As before, we used our estimated “central” population per species as the reference when presenting results, but results are very similar when different populations are used as reference. We also calculate *R*′*_xy_* for 0-fold degenerate, nonsynonymous sites, a measure which is normalized using putatively neutral 4-fold degenerate synonymous sites, by dividing *R_xy_* for 0-fold sites by *R_xy_* for 4-fold sites. The custom scripts used to calculate all summary statistics are available at: https://github.com/j-e-james/TreeDFEScripts.

### DFE Inference

The DFE was primarily inferred using polyDFE (Tataru et al. 2017; Tataru and Bataillon 2019). PolyDFE implements a likelihood-based approach, and simultaneously infers the DFE while also accounting for the effects of other distorters of the SFS such as demography and errors in SNP polarization through the incorporation of nuisance parameters (Eyre-Walker et al. 2006), which are inferred for each category of the SFS. This method requires the specification of a class of neutral and a class of non-neutral sites, for which we used 0- and 4-fold degenerate sites, thereby avoiding site-counting issues that arise with 2- and 3-fold degenerate sites. As no change in 4-fold degenerate sites results in a change in amino acid, they are the best proxy for neutrally evolving sites in coding DNA, although it is possible that there is selection on synonymous codon usage in the species (Duret 2002). We then inferred the neutral and non-neutral site frequency spectra across species and populations, after first projecting our data down to the same number of individuals to account for any sites with missing data. Our analyses were run on data projected down to 40 haplotypes, that is, 20 individuals. All scripts required for the processing of data, and the polyDFE input files used in this analysis, are available at (https://github.com/j-e-james/TreeDFEScripts). PolyDFE allows for the fitting of both deleterious-only DFEs and mixed DFEs, which account for the possible effects of beneficial mutations on DFE inference. From the DFE fitted for beneficial mutations, polyDFE is also able to estimate *α*, henceforth referred to as *α*_DFE_, the rate of adaptive molecular evolution. In polyDFE, this is calculated from the full DFE for beneficial mutations, however, this may inflate estimates of *α*_DFE_ due to the inclusion of beneficial mutations with very small selective effects. We, therefore, followed Galtier et al. (2016) by incorporating a lower bound of 5 for the population selection coefficients of positive mutations to be used in the calculation of α_DFE_, as implemented in polyDFE v. 2.0 (Tataru et al. 2017). This lower bound is arbitrary, and changing it will have an impact on the estimated value of α_DFE_. Finally, we note that we only use polymorphism data when running PolyDFE, to avoid having to make the assumption that the DFE is invariant between the ingroup and outgroup species. PolyDFE is able to estimate the deleterious DFE accurately without divergence information, and the inclusion of divergence data provides little or no improvement to estimates of the beneficial DFE (Tataru et al. 2017; Booker 2020).

To ensure that, as far as possible, our polyDFE runs explored the full range of parameter space when estimating the DFE, we ran polyDFE a minimum of five times per species, using different starting parameters for each run (see supplementary table S4, Supplementary Material online for details). Runs in which parameters were close to the edges of their permitted ranges were removed; we then assessed whether our runs reliably returned similar estimated DFE parameters and had a small gradient of the likelihood. As DFE estimation entails considerable uncertainty, we then ran polyDFE a further four times per species, initializing runs using both analytically estimated parameters and those parameters previously found to return the smallest gradient of the likelihood, fitting a different model per run: in model 1 we fit a full (deleterious and advantageous mutations) DFE, including an estimation of the rate of misidentification of the ancestral allele, ɛ_anc_; in model 2 we fit a full DFE, without including the estimation of ɛ_anc_; in model 3 we fit a deleterious mutation-only DFE; in model 4 we again fit a deleterious mutation-only DFE, but without including an estimation of ɛ_anc_. All models include an estimation of nuisance parameters, which account for the effects of demography. We then performed model averaging over the four models, as described in Muyle et al. (2021), such that models are weighted by their AICs to account for uncertainty in parameter estimation. We calculated AIC weights and generated bootstrap datasets using the R functions provided in polyDFE (Tataru et al. 2017), available at https://github.com/paula-tataru/polyDFE. All other scripts used to conduct these analyses are available at https://github.com/j-e-james/TreeDFEScripts.

PolyDFE v2.0 (Tataru and Bataillon 2019) enables the simultaneous fitting of DFEs to multiple datasets, allowing for model comparisons to assess whether models in which DFE parameters differ between datasets provide a significantly better fit than models in which the DFE parameters are shared between datasets. In situations in which we were interested in comparing models (e.g. comparing populations), we inferred a full DFE, including nuisance parameters and ɛ_anc_, allowing these parameters to vary between datasets, as recommended by Tataru and Bataillon (2019), to account for differences in ancestral identification error and demographic processes between comparisons.

### Statistical Analyses

We considered correlations between our inferred DFE parameters, life history traits, and population genetics summary statistics. All statistical analyses and plotting were conducted in R, using scripts available at https://github.com/j-e-james/TreeDFEScripts.

## Results

### Summary Statistics

Our dataset comprises polarized SNPs from approximately 3 Mb of targeted genome sequencing for six European tree species which were sampled broadly across their range, with approximately 25 individuals sequenced per location. For all populations across species (see “population” definition above), we calculated population genetic summary statistics to investigate the efficiency of selection across species and among populations within species.

**Fig. 1.**
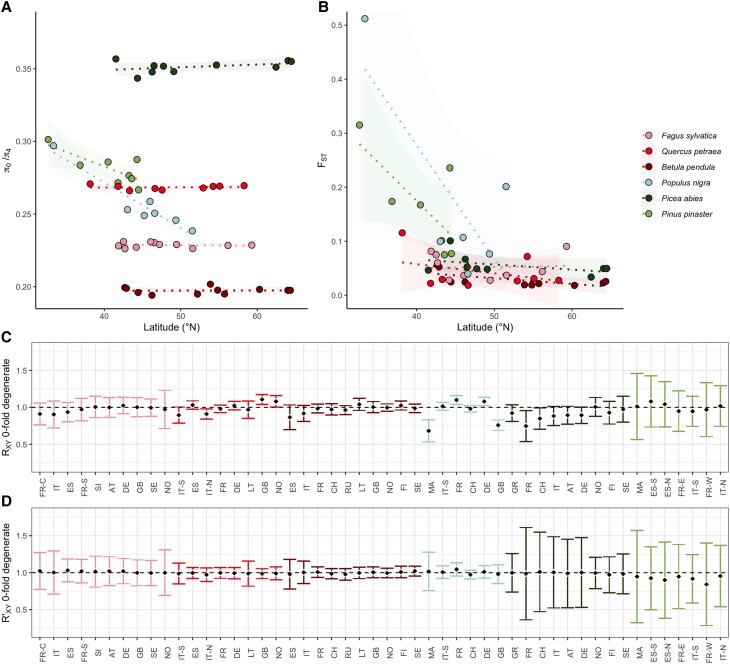
Levels of non-neutral diversity and mutation load are similar across populations within a species, despite different levels of population differentiation. The average *π*_0_/*π*_4_ per population for all species (A), and “focal” population pairwise *F*_ST_ (Wright's *F*_ST_) for all species (B), using a “central” (see Methods) population per species as a reference, plotted against average sampling latitude for the focal population. Lines shown are linear regression slopes, along with their 95% confidence intervals. In (C), we plot *R_xy_* for 0-fold degenerate, nonsynonymous sites, calculated per population, comparing focal populations (*x*) to a “central” (see Methods) reference population (*y*), while in (D), we plot *R*′*_xy_* for 0-fold degenerate, nonsynonymous sites, a measure which is normalized using putatively neutral 4-fold degenerate synonymous sites. In (C) and (D), black diamonds indicate the calculated values, while error bars are 95% confidence intervals on the estimate, calculated through jack-knifing. *X* axis labels are population codes, which begin with the two-letter country code of the sampling locations for each population, ordered by increasing latitude. The third letter provides additional location information for populations: C = Corsica, S = South, N = North, E = East, W = West (for exact sampling locations, see supplementary table S3, Supplementary Material online). Color codes for species, and species order, are consistent across all figure panels, with species ordered such that more closely related species are closer together.

Species vary broadly in *π*_0_/*π*_4_, the efficiency of purifying selection (Fig. 1*A*), with selection appearing to be comparatively inefficient in *P. pinaster* and *P. abies* relative to broad-leaved species such as *B. pendula*. This may reflect differences among species in effective population size. However, it does not clearly relate to levels of panmixia, despite the species exhibiting different degrees of genetic differentiation across their ranges (Fig. 1*B*). *B. pendula* exhibits very little population differentiation and has the most efficient selection of the six species. For *F. sylvatica*, *Q. petraea*, *B. pendula,* and *P. abies*, despite their broad geographic ranges, the efficiency of purifying selection was similar among populations within a species. By contrast, *P. nigra* and *P. pinaster* have the highest levels of population genetic structure, with strongly differentiated and isolated Moroccan populations, and there is a relationship with latitude and *π*_0_/*π*_4_ for both species (*P. nigra R*^2^ = 0.91, *P* = 0.0019 and *P. pinaster R*^2^ = 0.55, *P* = 0.04), with *π*_0_/*π*_4_ being lowest in populations at lower latitudes for both *P. nigra* and *P. pinaster*. However, these species have intermediate values of *π*_0_/*π*_4_ when comparing among species. *P. abies*, *F. sylvatica*, and *Q. petraea* show moderate levels of structure, with population *F*_ST_ increasing with latitude, and while *F. sylvatica* and *Q. petraea* have intermediate values of *π*_0_/*π*_4_, *P. abies* has the least efficient selection of any of the species studied.

It has been argued that metrics measuring the ratio of nonsynonymous to synonymous (or 0- to 4-fold degenerate) diversity are poor measures of genetic load (Do et al. 2015). We therefore also estimated the statistic *R_xy_*, which compares the frequency of derived alleles between a focal (*X*) and reference (*Y*) population. The neutral expectation is that the number of derived alleles is the same in the focal population as in the reference, while values of *R_xy_* above 1 indicate that the focal population has an excess of derived alleles.

Comparing focal populations to a single reference population (for which we used a population that was approximately central for the sampling locations per species, Fig. 1*C*), the most striking results are for *P. nigra* populations MA and GB, which show a deficit of derived alleles relative to the reference population. We also note a slight tendency for low latitude populations of *P. abies* to show a relative deficit of derived alleles, which agrees with our 0- to 4-fold diversity results. However, in the vast majority of populations, we find no deviation from the neutral expectation that the number of derived alleles at 0-fold degenerate sites is the same in the focal as in the reference population. If we use 4-fold degenerate synonymous sites to normalize *R_xy_* (*R*′*_xy_*, Fig. 1*D*), which has been suggested to account for the effects of population structure (Do et al. 2015; Grossen et al. 2020), we find that no population has a significant deficit relative to the focal population. Therefore, although population structure has resulted in a deficit or excess of mutations in some populations, there is little evidence that populations differ in their genetic load.

### Species DFE

**Fig. 2.**
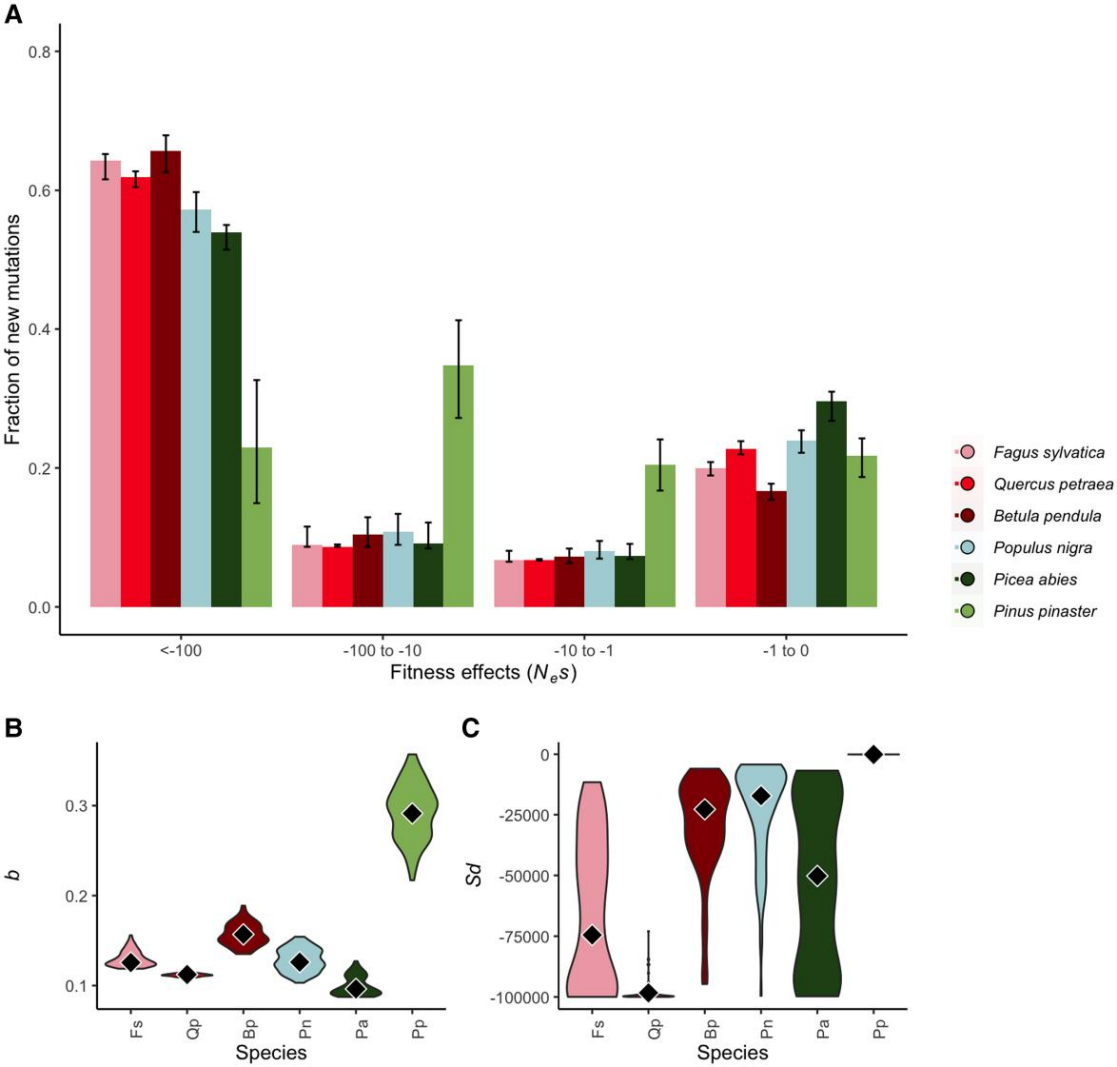
Species differences in the deleterious-only DFE. (A) Shows the model-averaged discretized DFE, that is, the fraction of new mutations in each scaled fitness effect (*N_e_s*) category. Black bars indicate 95% confidence intervals on the estimated fraction, as estimated from model-averaged bootstrap replicates. (B) Violin plots of the shape parameter, *b*, and (C) Violin plots of the scale parameter *S_d_*, for the gamma distribution of deleterious fitness effects per species. Black diamonds are the inferred model-averaged parameters, while violins show the 95% confidence intervals, as estimated from model-averaged bootstrap replicates.

**Fig. 3.**
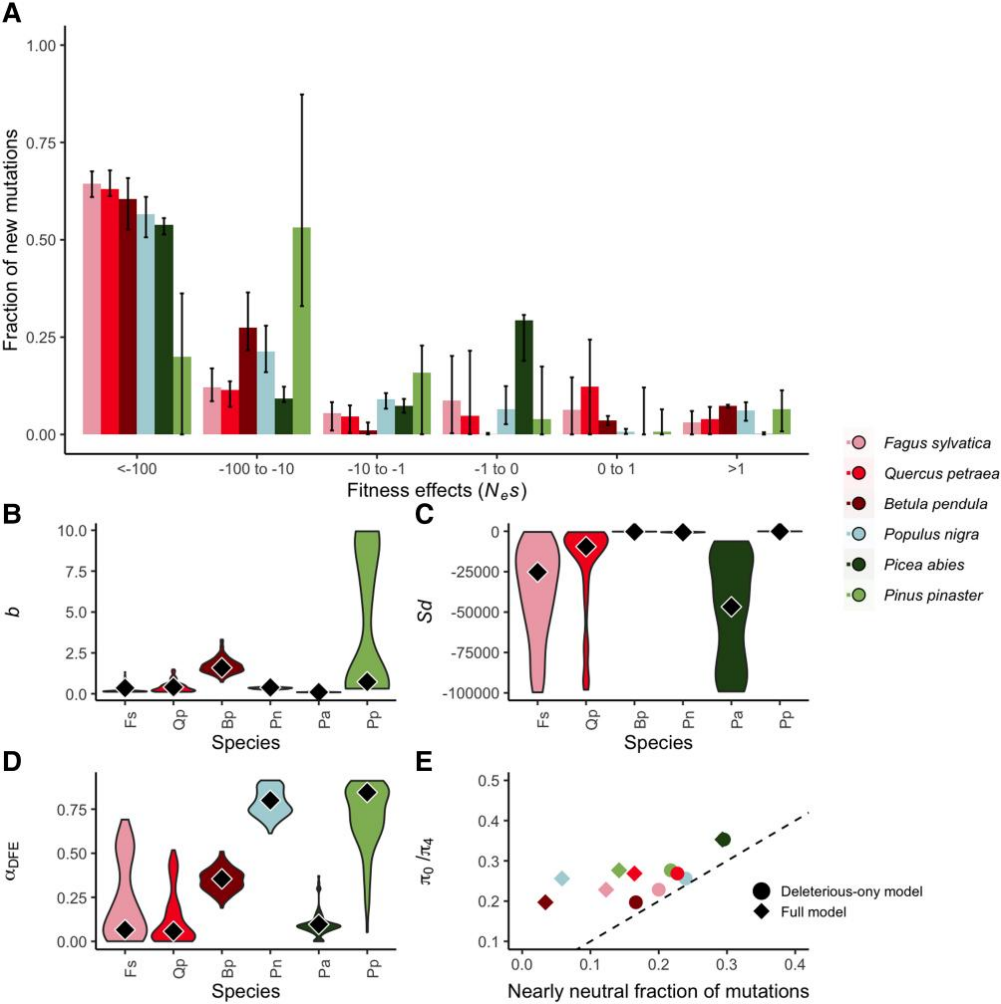
Species differences in the full DFE. (A) Shows the model-averaged discretized DFE, that is, the fraction of new mutations in each scaled fitness effect (*N_e_s*) category. Black bars indicate 95% confidence intervals on the estimated fraction, as estimated from model-averaged bootstrap replicates. (B) Violin plots show the shape parameter, *b*, the scale parameter, (C) *S_d_*, for the gamma distribution of deleterious fitness effects per species, (D) α_DFE_, the estimated fraction of substitutions inferred to be adaptive. Black diamonds are the inferred model-averaged parameters, while violins show the 95% confidence intervals, as estimated from model-averaged bootstrap replicates. In (E), we show the fraction of slightly deleterious (−1 < *N_e_s* < 0) mutations plotted against the ratio of 0- to 4-fold degenerate nucleotide diversity. Circles represent the fraction as inferred from the deleterious-only DFE model, diamonds represent the fraction as inferred from the full (advantageous and deleterious) DFE model. The dashed line indicates *x* = *y*. For *P. abies*, the diamond and circle overlap.

We inferred the full DFE for all species, incorporating a gamma-distributed deleterious DFE and an exponential-distributed beneficial DFE, using only polymorphism data (Tataru et al. 2017). The gamma distribution is a flexible and commonly used distribution, and is parameterized by two values: the shape parameter, *b*, which is inversely related to the coefficient of variation of the strengths of selection acting on new mutations, and the scale parameter, *S_d_*, which is the mean scaled strength of selection (*N_e_s*) acting on new mutations. We also inferred the purely deleterious DFE for all six species to assess whether incorporating beneficial mutations improves our DFE model inference (Fig. 2). We fitted the full DFE and the deleterious-only DFE models both with and without incorporating an estimation of the error rate for the inference of the ancestral state of alleles, ɛ_anc_, and conducted a model averaging procedure (see Methods and supplementary fig. S2, Supplementary Material online to see the fit of each model, and see supplementary table S5, Supplementary Material online for all model-averaged inferred parameters for the deleterious and beneficial DFEs), such that the estimated DFE parameters presented here incorporate the degree of model uncertainty (Fig. 3; Table 1). In three of the species in our dataset, incorporating the rate of ancestral misidentification did not significantly improve the fit of the DFE model; while in *F. sylvatica*, *Q. petraea* and *P. abies* we see a small model improvement (*P*-values of likelihood ratio tests comparing models are 0.018, 0.016, and 0.0039, respectively); these species have high *S_d_* values and a high proportion of adaptive substitutions, which is the selective regime in which we expect the rate of ancestral error to be inflated in polyDFE analyses (Tataru et al. 2017). Generally, the species in our dataset have similar values of *b*, but vary considerably in *S_d_*. However, *S_d_* values should be interpreted with caution, because *S_d_* is not related to the distribution of selection coefficients of segregating mutations in a straightforward way (see supplementary fig. S3, Supplementary Material online for details).

**Table 1:** Model-averaged estimates of the DFE parameters, for all species

Species	*S_d_*	*b*	Fraction of mutations −1 < *N_e_s* < 0	*π*_0_/*π*_4_	Model
*Fagus sylvatica*	−25,000	0.36	0.20	0.23	− ɛ_anc_
*Quercus petraea*	−9500	0.41	0.23	0.27	+− ɛ_anc_
*Betula pendula*	−190	1.59	0.17	0.20	+−
*Populus nigra*	−571	0.39	0.24	0.26	+−
*Picea abies*	−47,000	0.097	0.30	0.35	− ɛ_anc_
*Pinus pinaster*	−64	0.73	0.22	0.28	+−

*S_d_*: the mean scaled strength of deleterious selection acting on new mutations rounded to two S. F., that is, the scale parameter of the gamma-shaped deleterious DFE; *b*: the shape parameter of the gamma-shaped deleterious DFE, which is inversely related to the coefficient of variation in the fitness effects of new deleterious mutations; the inferred fraction of mutations with fitness effects between −1 and 0, that is, the nearly neutral fraction of slightly deleterious mutations; and *π*_0_/*π*_4_. DFE parameters shown are model-averaged, such that estimates are weighted by model AIC. The best model, as ascertained using likelihood ratio tests, is indicated in the last column; whether fitting a deleterious-only DFE (−) or a full DFE including beneficial mutations (+−), and whether including the rate of error in the inference of the ancestral state improves the model fit (ɛ_anc_).

In five of the six species, a model incorporating beneficial mutations into estimates of the DFE was the most highly weighted, although only in four species was this model a significantly better fit to the data. Ignoring the contribution of beneficial mutations to the DFE in these species leads to a reduction in the inferred value of *b* and to an increase in the inferred value of *S_d_* (Figs. 2 and 3). We used polyDFE to estimate the rate of adaptive molecular evolution (i.e. the proportion of nonsynonymous substitutions that are beneficial), α_DFE_, incorporating a lower bound for the minimum strength of selection acting on new mutations. We demonstrate the effects of different bounds on the estimate of α_DFE_ in see supplementary fig. S4, Supplementary Material online. We find that the rate of adaptive evolution is fairly high in some of the tree species, particularly in *B. pendula* and *P. nigra*, suggesting that adaptive substitutions are common in these forest tree species (Fig. 3).

*P. abies* and *P. pinaster,* the two conifer species included in this study, are an interesting pair to compare. *P. abies* has remarkably low values of α_DFE_, which is particularly surprising given the fairly high inferred α_DFE_ in the other conifer in the dataset, *P. pinaster*. These differences may arise due to *P. pinaster* having relatively differentiated populations, which could facilitate local adaptation due to the limited influx of alleles from other populations, whereas *P. abies* has less population structure, that is, greater levels of admixture, and uniformly high levels of purifying selection across its range (Figs. 1 and 3). It is also interesting to note that *P. pinaster* has quite a distinct discretized DFE compared to the other species in the dataset, with a high inferred *b*, a low inferred absolute *S_d_*, and a relatively small estimated fraction of mutations falling into the most strongly deleterious category (*N_e_s* < −100). *P. pinaster* has fewer SNPs compared to the other species in the dataset, and so we have less confidence in these results, however, these differences could represent the greater phylogenetic distance between *P. pinaster* with the other forest trees in the dataset. The most closely phylogenetically related species in this dataset are *F. sylvatica* and *Q. petraea*, which do have similar DFEs (shown in Figs. 2 and 3). However, we find that for these two species, DFE models that are fitted independently per species have significantly better log-likelihoods than models in which either both *b* and *S_d_* are shared between species (*P* = 0.05), or models in which only *b* is shared between species (*P* = 0.03), as might be the case if the two species shared a DFE but had different effective population sizes.

### Drivers of Differences in the DFE at the Species Level

#### GC-biased Gene Conversion

It has been demonstrated that GC-biased gene conversion can result in misinference of the DFE (Bolívar et al. 2018). CpG sites are highly mutable, and prone to polarization error. We therefore repeated our analyses restricting our dataset to GC-conservative mutations (see supplementary table S6 and fig. S5, Supplementary Material online for details). We found that fitting the DFE parameters independently for GC-conservative mutations does not provide a better model fit than allowing DFE parameters to be shared between GC-conservative mutations and the full SNP dataset. Our inferred DFEs are similar across datasets (see supplementary figs S6 and S7, Supplementary Material online for details), it is therefore unlikely that differences in GC-biased gene conversion, due, for example, to differences in recombination rate among species, explain differences in the DFE among species.

#### Life History Traits and *N_e_*

There are no significant correlations between any of the estimated parameters of the DFE and the two life history traits that we tested, maximum longevity and age at first reproduction (i.e. minimum age at flowering), which were previously shown to predict genetic diversity and the efficiency of selection in plants (Chen et al. 2017). However, the mean scaled strength of selection acting on deleterious variants, *S_d_*, varies across species, increasing with a proxy of *N_e_*, the level of neutral nucleotide site diversity *π*_4_, which reflects the stronger effect of drift in smaller populations, as expected under the Nearly Neutral Theory (Spearman's *rho* = −0.79, *P* = 0.048, Pearson's *R* = 0.72, *P* = 0.065).

As expected under the Nearly Neutral Theory, the fraction of mutations that we infer to be nearly neutral from the DFE is correlated to our estimate of *π*_0_/*π*_4_ (Fig. 2*E*). However, *π*_0_/*π*_4_ is always greater than the nearly neutral fraction of mutations as estimated from the DFE. This is likely to be due to the contribution of segregating beneficial and slightly beneficial mutations to diversity in these species. Indeed, if we consider results from models in which we fit the deleterious DFE only (Fig. 2*E*), this systematic difference between *π*_0_/*π*_4_ and the nearly neutral fraction is reduced. *B. pendula* and *P. nigra* are particular outliers, highlighting the effect that beneficial variants have on patterns of molecular evolution in these species.

#### Gene Content

The differences in the DFE that we observe between species are unlikely to be due to differences in gene content, or differences in genes sequenced, between species. Indeed, the parameters of the DFE were very similar when calculated across all genes in the dataset, and when calculated only for those common orthologs that were sequenced in all six species (for details of the relative proportions of all-species orthologs, see supplementary table S7, Supplementary Material online for details). Only in *P*. *pinaster* do likelihood ratio tests suggest that an independent DFE for orthologs found in all species is a better fit to the data than a shared DFE for all genes. We found that a slightly higher fraction of new mutations is inferred to be strongly deleterious in orthologs (Fig. 3), which we might expect as such genes are likely to be older, involved in many important biological functions, and under strong purifying selection. This suggests that in *P. pinaster*, genes in our dataset that are not part of the all-species ortholog set might experience differences in selective effects; they may be under less strong purifying selection. We also note a lower fraction of adaptive substitutions in all-species orthologs.

### Differences Among Populations Within Species

The DFE is similar across populations of the same species, with species explaining a considerable proportion of the variation in the parameters of the deleterious and beneficial DFE as calculated across populations (Fig. 5; for deleterious-only DFE inferences see supplementary fig S8, Supplementary Material online for details). For the majority of populations, we could not reject the null model that the DFE of the population is the same as the DFE inferred for the species as a whole. This was true even under a very conservative scenario in which we fit models assuming that both *b* and *S_d_* are shared across the populations and the species on average. In other words, the mean scaled strength of selection and the variance in fitness due to new mutations is consistent across populations, despite any differences in demographic history and local adaptation to environmental conditions between populations.

**Fig. 4.**
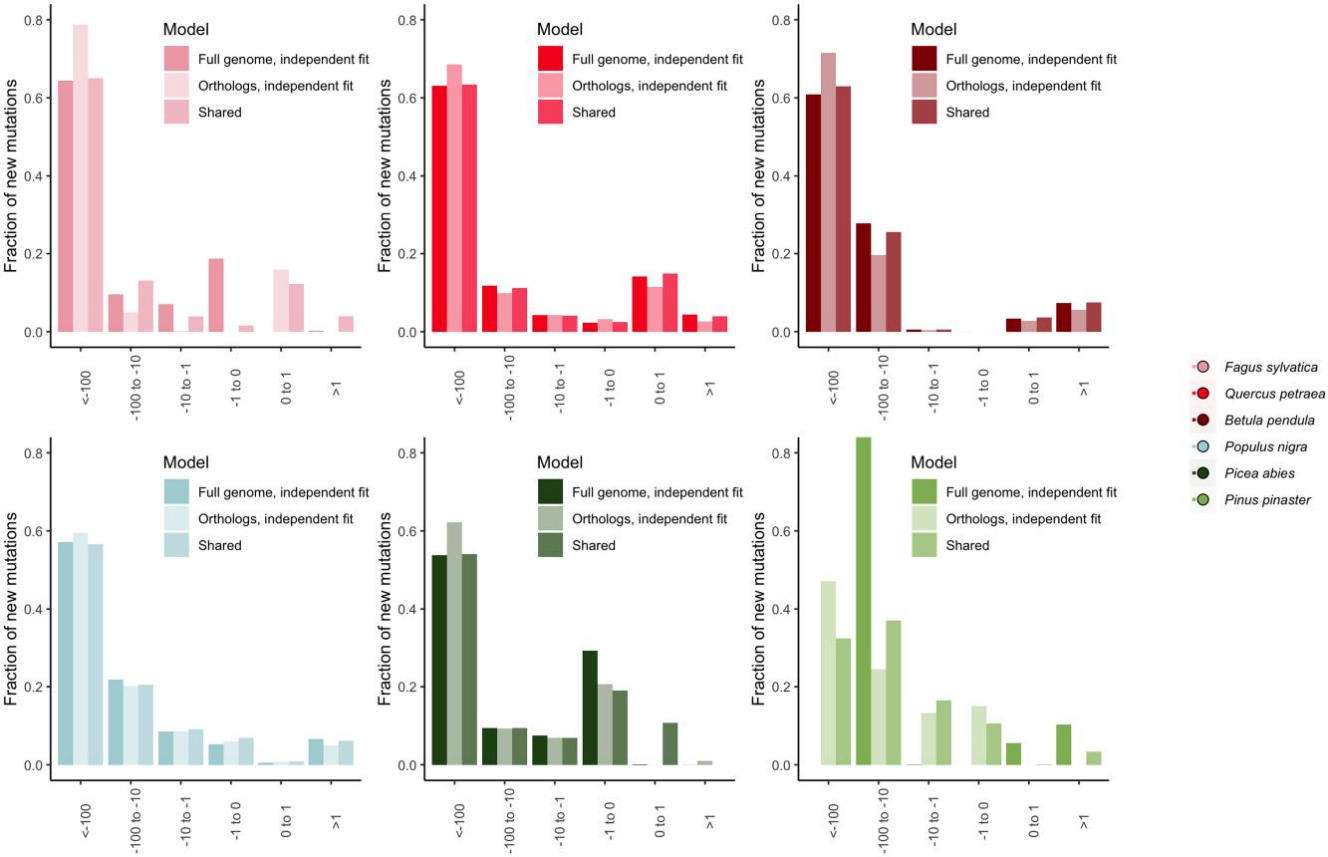
Discretized DFEs for each species, showing model comparisons for different categories of genes. Darkest bars show the independent fit for all genes, lightest bars show the independent fit for orthologs found in all species, intermediate bar shows the fit if the parameters are inferred to be shared across the all-species orthologs and the full dataset. We show model fits for the full DFE, including an estimate of the rate of ancestral allele misidentification, ɛ_anc_, for all species.

**Fig. 5.**
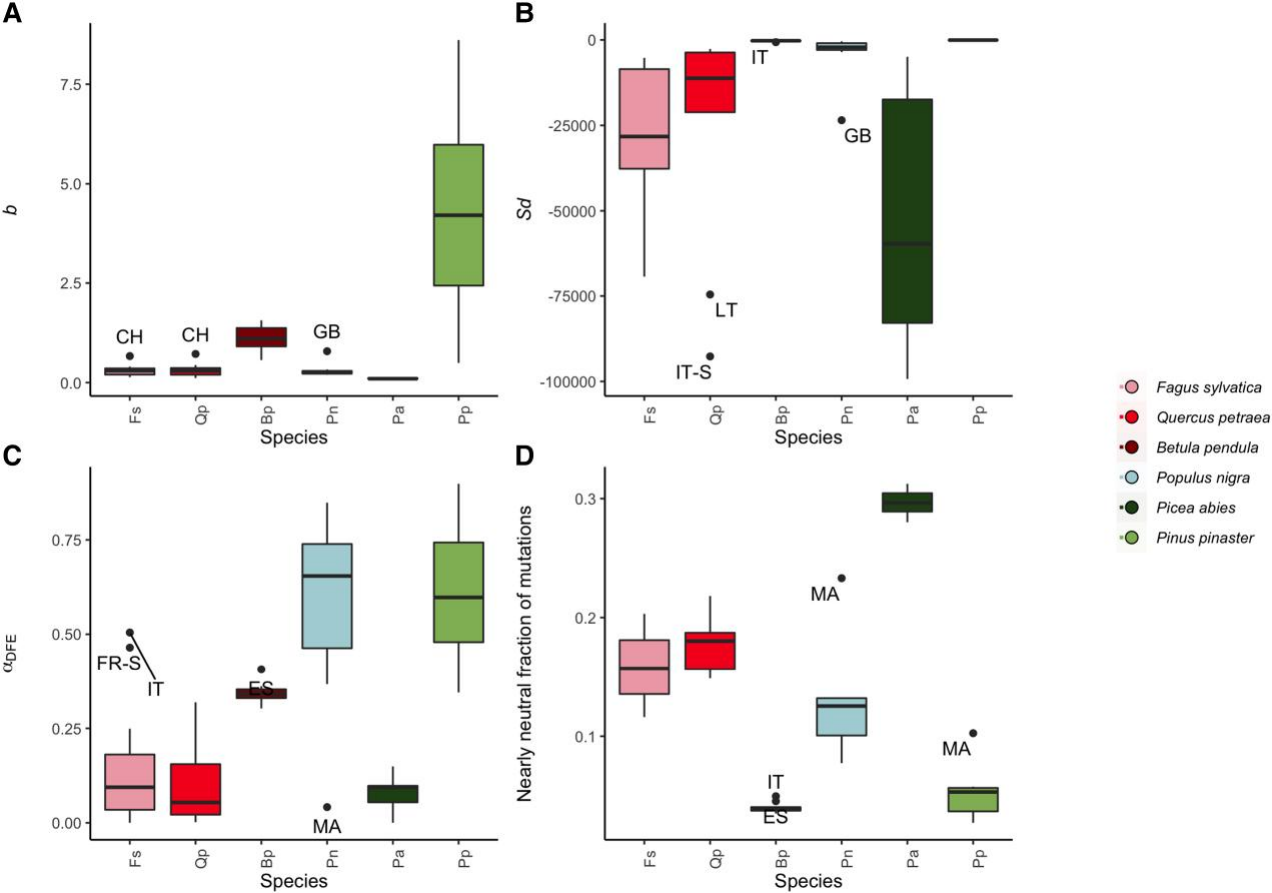
DFE parameters are consistent across populations within a species for the full DFE. Shown are the model-averaged inferred parameters. We plot the shape (A) and scale (B) parameter of the gamma deleterious distribution of fitness effects, (C) α_DFE_, the proportion of substitutions that are expected to be adaptive, (D) the proportion of mutations inferred to be effectively neutral, that is, the fraction of mutations for which −1 < *N_e_s* < 1. Boxplots show the distribution of values per species, with outlier points indicated as black dots, and labeled by their population codes. Population codes always start with two letter country codes, S = South. For exact sampling locations, see supplementary table S3, Supplementary Material online.

There are some exceptions to these general trends. We note that while results for *P. pinaster* populations indicate a considerably greater spread of inferred *b* among populations (Fig. 5*A*), no differences between populations are statistically supported. However, model comparison results indicate that two *B. pendula* populations might have different DFEs from the species on average (ES and IT, see supplementary fig S9, Supplementary Material online for details). We also find that one *Q. petraea* population (LT), four *F. sylvatica* populations (AT, GB, NO, and SI, see supplementary fig S9, Supplementary Material online for details) and three *P. nigra* populations (GB, ITS, and MA, see supplementary fig S9, Supplementary Material online for details), have significantly different DFEs from the DFE as calculated over all populations of each species (Fig. 4). Our results suggest both *S_d_* and *b* differ between these populations and the dataset as a whole. We note that of these outlier populations only two, *F. sylvatica* NO and *P. nigra* GB, are significant after performing a strict Bonferroni correction for multiple testing.

In these analyses, we compare a population-specific DFE to a species-level DFE inferred over all populations, which might reduce differences between populations and the species-level “pooled” DFE. We therefore repeated our analyses, comparing each focal population to a central reference population. We again found little difference in the DFE between populations within a species. The only populations that had significantly different DFEs to the central population were *F. sylvatica* NO and *P. nigra* MA and GB, and thus we can conclude that our results are consistent.

### Drivers of Differences in the DFE at the Population Level

At the species level, evidence of a relationship between population differentiation and variation in the effectiveness of selection, or in the shape of the DFE, is not clear. *P. pinaster*, *P. abies,* and *P. nigra*, have higher mean *F*_ST_ values by approximately an order of magnitude, however, among-population variation in parameters of the DFE and the estimated effectiveness of selection are similar for both these and other species in the dataset with lower levels of population differentiation (see Fig. 4).

To investigate more systematically the possibility that differentiation leads to differences in the DFE, we correlated population pairwise *F*_ST_ with population differences in the DFE parameters *S_d_* and *b*, and population differences in *π*_0_/*π*_4_. Although in some species, greater population differentiation appears to correlate with larger differences in parameters of the DFE, this relationship is not consistent (see supplementary fig S10, Supplementary Material online for details).

## Discussion

Here, we have shown that both the efficiency of selection and the DFE differ among species, but that there is relatively little variation among populations within species. Our results suggest striking differences between different tree species, with conifers generally having a smaller fraction of highly deleterious mutations. This variation is not driven by differences in gene content between species. The nature of our exome capture dataset has resulted in a dataset that contains a high proportion of genes which are orthologs, with only 140 out of a total of 1,042 genes sequenced in only a single species. Even when we restrict our analysis to orthologous genes sequenced across every species, which constituted an average of 32% of genes sequenced per species (ranging from 26% in *P. nigra* to 38% in *F. sylvatica*), differences between species in both the mean scaled strength of selection (*S_d_*) and the coefficient of variation in the strength of selection (*b*) remain the same.

An important caveat of the present study is that in order to estimate the DFE, we have assumed that the DFE can be reasonably well approximated as a continuous gamma distribution. This allowed us to conduct straightforward comparative analyses across species and populations. However, it is important to acknowledge that although the DFE is commonly modeled as a gamma distribution (Bataillon and Bailey 2014; Martin and Lenormand 2006), other distributions can be theoretically justified (Loewe and Charlesworth 2006), and in some studies better support has been found for alternative distributions such as the lognormal or multimodal (Kousathanas and Keightley 2013; Loewe and Charlesworth 2006; Sawyer et al. 2003). Alternative distributions may be better able to model high concentrations of strongly deleterious or lethal mutations more accurately, a feature that has been observed in some mutation accumulation experiments (Eyre-Walker and Keightley 2007). Such mutations have little chance of being observed in most datasets due to their rarity, and as such the shape of the most deleterious class of mutations is based on projecting from the DFE. However, previous analyses have found that the inferred parameters of the gamma DFE are not greatly affected by including or excluding an additional parameter that takes these most deleterious mutations into account (Eyre-Walker et al. 2006). At the other end of the selective scale, alternative models may also be better able to cope with the fact that it is difficult to examine the DFE for mutations that are either neutral or have very small selection coefficients (Welch et al. 2008). Some studies have considered models that consist of a distribution of selected effects plus a point mass of neutral mutations, which have sometimes been found to fit data well (Loewe and Charlesworth 2006; Kim et al. 2017).

Our analysis was conducted on targeted resequencing data. This approach allowed for the sampling of a high number of individuals per population and per species, increasing the amount of power we had to infer DFE's parameters, which are notoriously difficult to estimate. While it is possible that the genomic regions used in this analysis do not reflect processes across the genome, a dataset restricted to all-species orthologs has a similar DFE to the dataset as a whole, making it likely that our DFEs are representative of the whole coding genomes of the species included in this study. Interestingly, recent work (Simons et al. 2022) has argued that in a scenario in which most traits are highly polygenic and experiencing stabilizing selection, the distribution of selection coefficients will be similar across loci that underlie all such traits. The orthologous genes which make up the majority of the coding sequences included in this analysis are perhaps likely to experience both stabilizing selection, and to underlie traits that are highly polygenic, and hence be well described by the model developed by Simons et al.

Variation in the efficiency of selection at the among-population level in the species in our dataset is low. It is perhaps not surprising that many populations have highly similar DFEs to that inferred for the species overall, given the remarkably similar levels of *π*_0_/*π*_4_ across populations in most species (Fig. 1), and their often similar demographic histories. Although many of the species are important economically, their use by humans is unlikely to have affected the DFE, especially given that the domestication of other crop plants has had little effect on their DFEs compared to their wild relatives (Chen et al. 2017). Comparatively, forest tree domestication and breeding is in its infancy, and the increasing effects of human activity have not yet had sufficient time to have a large impact on the tree species in this study. Recent work on the demographic histories of these species found that populations were remarkably stable in recent time, with little detectible effective population size reductions even in the face of periods of glaciation (Milesi et al. 2023). The two populations for which we have the strongest evidence for differences in the DFE, on the other hand, are somewhat unusual in terms of their demographic histories. The *P. nigra* GB population experienced a sharp population decrease in the past, and subsequently recovered. *F. sylvatica* NO also experienced a fairly extreme decrease in *N_e_*, from which it has since recovered. Both populations differ from the species as a whole in that they have a comparatively high fraction of strongly deleterious mutations.

It has been suggested that differences in genetic load among populations might drive differences in the DFE and that populations at the edge of a species’ range will have a temporarily increased mutation load relative to central populations, due to the increased importance of drift in these populations (Peischl et al. 2013; Willi et al. 2018). While for most populations we find no evidence that mutation load differs between populations, in two *P. nigra* populations, GB and MA, there is a reduction in the proportion of derived alleles relative to other *P. nigra* populations. *P. nigra* is also one of the two species that show a relationship between the efficiency of selection and latitude. For the GB population, greater purging of deleterious derived alleles is in line with our finding that a high fraction of new mutations in this population are strongly deleterious, and that the mean strength of selection acting on new deleterious mutations is greater.

However, for the Moroccan (MA) *P. nigra* population, a comparatively low fraction of new mutations is inferred to be strongly deleterious (see supplementary fig. S9, Supplementary Material online for details). This population is differentiated, and in addition, there is little correlation in the frequency of alleles between this and other *P. nigra* populations. There are also a number of fixed differences between MA and other *P. nigra* populations. The DFE might differ due to these fixed differences; for example, new mutations may be less strongly deleterious when they occur on a genetic background in which many deleterious mutations are already present. However, the differences we observe are not due to inbreeding; we do not see any evidence for a shift in mating system in the MA population. There are no clonal individuals in the MA population, nor any increase in the degree of relatedness between individuals (as estimated via the KING algorithm, implemented in PLINK; Manichaikul et al. 2010; Chang et al. 2015).

The fact that we generally do not find evidence for variation in the DFE at the population level does not mean that there is no local adaptation occurring in response to different environmental conditions across populations. Tree species generally show high levels of local adaptation, for example, for phenological traits (Savolainen et al. 2007), and the species in this study were generally inferred to have a high proportion of beneficial substitutions, with the exception of *P. abies* (α_DFE_, Fig. 3*C*). Infrequent, strong selective sweeps are expected to leave little signature on the SFS (Booker 2020), and thus have a relatively small effect on statistics calculated from it, including the DFE. Therefore, it is possible that the tree populations do experience local adaptation through selective sweeps, the effects of which we will not detect with the summary statistics considered here. However, the DFE is informative about the strength of purifying selection and the variance of mutational effects, which do not differ among populations in the tree species in this study.

It has been hypothesized that higher population differentiation might lead to greater differences in the parameters of the DFE between populations. Our general finding is that there is some relationship between population differentiation and differences in the DFE, particularly in the strength of deleterious selection (see supplementary fig. 10, Supplementary Material online for details), but it is not consistent. It is interesting to consider this finding in light of the scattering and collecting phase of the coalescent (Wakeley 1999). During the collecting phase, the more ancient part of a species’ history, the rate of coalescence is independent of the current geographic distribution of individuals. However, demographic history and geography will determine coalescence during the more recent part of a species history, the scattering phase. From this study, it seems that the DFE is more strongly affected by ancient events, that is, the collecting phase of the coalescent, and the long-term *N_e_*, leading to similar strengths of purifying selection across most populations of the same species. Whether this finding is generally true remains to be seen; the tree species in this study have moderate to high dispersal rates, however, stronger patterns of isolation by distance will lead to a stronger signal during the scattering phase (Wilkins 2004), which may result in the scattering phase having a greater impact on the DFE.

Why do differences in the DFE exist at the species level? Neither of the life history traits that we examined, maximum longevity and average age at first flowering, showed a relationship with any parameters of the DFE. We focussed on these two traits as they have been previously shown to be predictive of genetic diversity in plants (Chen et al. 2017), although it is possible that other life history traits might affect the DFE. Previous work suggests that there might be a relationship between the DFE and large life history changes, such as transitioning from selfing to outcrossing. For example, in the herb *Arabis alpina*, selfing was associated with a reduction in the fraction of mutations inferred to experience strong negative selection, and a general reduction in the efficiency of purifying selection, while populations with mixed mating systems had very similar DFEs to outcrossing populations, with no signal of increased genetic load (Laenen et al. 2018). Relatedness also clearly plays a part- previous studies on closely related species have found that they share the same DFE (Chen et al. 2017; Castellano et al. 2019; Liu et al. 2022). The most closely related species in our dataset, *F. sylvatica* and *Q. petraea*, also appear to have more similar DFEs (see, for example, Fig. 3), although model comparison tests indicate that fitting DFEs independently to these species provides a better fit to the data, albeit only slightly (log-likelihoods of independent and shared models: −523.6194, −526.7272, *P* = 0.045). It may be that some slow evolving aspect of genome biology, for example, gene interaction networks, methods of gene expression regulation, or genome organization or size, eventually lead to differences in DFEs between species. The possibility that genome organization could affect the DFE was previously investigated by Hämälä and Tiffin (2020), who showed that a number of genome features could influence selective constraint, including expression level, expression variability, and gene network connectivity, while Castellano et al. (2020), found that gene density was negatively correlated to nonsynonymous diversity, possibly due to greater constraint acting on gene dense regions. This is of particular relevance to the species included in this study, because conifer genomes are considerably larger than the genomes of other tree species (De La Torre et al. 2014).

In summary, genome and species biology are important determinants of the DFE, whose long-term effects dominate short-term processes. Our findings indicate that despite differences among populations in environmental challenges faced, the mean strength of selection experienced by new mutations and their variation in selective effects remain similar across populations. The DFEs of the tree species in this study are stable, reflecting deep processes. A large change, such as a shift in breeding system, for example, from outcrossing to inbreeding, or genome structure, may be required before the DFE differs between populations or species.

GenTree Consortium: Paraskevi Alizoti^1^, Ricardo Alía^2^, Olivier Ambrosio^3^, Filippos A Aravanopoulos^1^, Georg von Arx^4^, Albet Audrey^5^, Francisco Auñón^2^, Camilla Avanzi^6^, Evangelia Avramidou^1^, Francesca Bagnoli^7^, Marko Bajc^8^, Eduardo Ballesteros^2^, Evangelos Barbas^1^, José M García del Barrio^2^, Cristina C Bastias^9^, Catherine Bastien^10^, Giorgia Beffa^11^, Raquel Benavides^12^, Vanina Benoit^13^, Frédéric Bernier^5^, Henri Bignalet^5^, Guillaume Bodineau^14^, Damien Bouic^5^, Sabine Brodbeck^11^, William Brunetto^15^, Jurata Buchovska^16^, Corinne Buret^13^, Melanie Buy^17^, Ana M Cabanillas-Saldaña^18^, Bárbara Carvalho^12^, Stephen Cavers^19^, Fernando Del Caño^2^, Sandra Cervantes^20,21^, Nicolas Cheval^5^, José M Climent^2^, Marianne Correard^22^, Eva Cremer^23^, Darius Danusevičius^16^, Benjamin Dauphin^24^, Jean-Luc Denou^5^, Bernard Dokhelar^5^, Alexis Ducousso^25^, Bruno Fady^26^, Patricia Faivre-Rampant^27^, Anna-Maria Farsakoglou^1^, Patrick Fonti^4^, Ioannis Ganopoulos^28^, Olivier Gilg^22^, Nicolas De Girardi^29^, René Graf^11^, Alan Gray^30^, Delphine Grivet^31^, Felix Gugerli^24^, Christoph Hartleitner^32^, Katrin Heer^33^, Enja Hollenbach^34^, Agathe Hurel^25^, Bernard Issenhuth^5^, Florence Jean^15^, Véronique Jorge^35^, Arnaud Jouineau^36^, Jan-Philipp Kappner^34^, Robert Kesälahti^37^, Florian Knutzen^23^, Sonja T Kujala^38^, Timo A Kumpula^37^, Katri Kärkkäinen^38^, Mariaceleste Labriola^39^, Celine Lalanne^25^, Johannes Lambertz^34^, Gregoire Le-Provost^25^, Vincent Lejeune^14^, Isabelle Lesur-Kupin^40,41^, Joseph Levillain^42^, Mirko Liesebach^43^, David López-Quiroga^12^, Ermioni Malliarou^1^, Jérémy Marchon^11^, Nicolas Mariotte^36^, Antonio Mas^12^, Silvia Matesanz^44^, Benjamin Meier^11^, Helge Meischner^34^, Célia Michotey^17^, Sandro Morganti^11^, Tor Myking^45^, Daniel Nievergelt^4^, Anne Eskild Nilsen^45^, Eduardo Notivol^46^, Dario I. Ojeda^47^, Sanna Olsson^31^, Lars Opgenoorth^24,48^, Geir Ostreng^45^, Birte Pakull^43^, Annika Perry^30^, Sara Pinosio^7,49^, Andrea Piotti^6^, Christophe Plomion^40^, Nicolas Poinot^5^, Mehdi Pringarbe^22^, Luc Puzos^5^, Annie Raffin^5^, José A Ramírez-Valiente^2^, Christian Rellstab^24^, Dourthe Remi^5^, Oliver Reutimann^11^, Sebastian Richter^34^, Juan J Robledo-Arnuncio^2^, Odile Rogier^35^, Elisabet Martínez Sancho^4^, Outi Savolainen^37^, Simone Scalabrin^50^, Volker Schneck^51^, Silvio Schueler^52^, Ivan Scotti^26^, Sergio San Segundo^2^, Vladimir Semerikov^53^, Lenka Slámová^4^, Ilaria Spanu^54^, Jørn Henrik Sønstebø^45^, Jean Thevenet^22^, Mari Mette Tollefsrud^45^, Norbert Turion^22^, Fernando Valladares^12^, Giovanni G. Vendramin^7^, Marc Villar^55^, Marjana Westergren^56^, Johan Westin^57^

^1^Aristotle University of Thessaloniki, School of Forestry and Natural Environment, Laboratory of Forest Genetics and Tree Improvement, 541

^2^Instituto Nacional de Investigación y Tecnología Agraria y Alimentaria—Centro de Investigación Forestal (INIA-CIFOR), Ctra. de la Coruña km 7.5, 28040, Madrid, Spain

^3^INRAE, URFM F-84914, Avignon, France

^4^Forest Dynamics, Swiss Federal Research Institute WSL, 8903 Birmensdorf, Switzerland

^5^INRAE, UEFP, F-33610, Cestas, France

^6^Institute of Biosciences and Bioresources, National Reaseach Council of Italy

^7^Institute of Biosciences and Bioresources, National Reasearch Council of Italy (IBBR-CNR), 50019 Sesto Fiorentino, Italy

^8^Slovenian Forestry Institute, Vecna pot 2, 1000 Ljubljana, Slovenia

^9^Centre d’Ecologie Fonctionnelle et Evolutive (CEFE), CNRS, UMR 51

^10^INRAE, Dept ECODIV, F-45075, Orléans, France

^11^Biodiversity & Conservation Biology, Swiss Federal Research Institute WSL, 8

^12^LINCGlobal, Department of Biogeography and Global Change, Museo Nacional de Ciencias Naturales, CSIC, Serrano

^13^INRAE, ONF, BioForA, F-45075, Orléans, France

^14^INRAE, GBFOR, F-45075, Orléans, France

^15^INRAE, URFM, F-849

^16^Vytautas Magnus University, Studentu Street 11, 53361, Akademija, Lithuania

^17^INRAE, URGI, F-78026, Versailles, France

^18^Departamento de Agricultura, Ganadería y Medio Ambiente, Gobierno de Aragón, P. Mª Agustín 36, 50071, Zaragoza, Spain

^19^UK Centre for Ecology & Hydrology (UKCEH), EH26 0QB Bush Estate, United Kingdom

^20^Department of Ecology and Genetics, University of Oulu, 90014 Oulu, Finland

^21^Biocenter Oulu, University of Oulu, 90014 Oulu, Finland

^22^INRAE, UEFM, F-84914, Avignon, France

^23^Bavarian Institute for Forest Genetics, Forstamtsplatz 1, 83317, Teisendorf, Germany

^24^Biodiversity and Conservation Biology, Swiss Federal Research Institute WSL, 8903 Birmensdorf, Switzerland

^25^INRAE, Université de Bordeaux, BIOGECO, F-33770, Cestas, France

^26^National Research Institute for Agriculture, Food and the Environment (INRAE), 84914 Avignon, France

^27^University of Paris-Saclay, INRAE, Study of Plant Genome Polymorphism, 91000 Evry-Cour-couronnes, France

^28^Institute of Plant Breeding and Genetic Resources, Hellenic Agricultural Organization DEMETER (ex NAGREF), 57001, Thermi, Greece

^29^Swiss Federal Research Institute WSL, 8903 Birmensdorf, Switzerland

^30^UK Centre for Ecology and Hydrology, Bush Estate Penicuik, EH26 0QB, Edinburgh, UK

^31^Institute of Forest Sciences (ICIFOR-INIA), CSIC, 28040 Madrid, Spain

^32^LIECO GmbH & Co KG

^33^Forest Genetics, Albert-Ludwigs Universität Freiburg, Bertoldstraße 17, 79098 Freiburg, Germany

^34^Philipps University Marburg, Faculty of Biology, Plant Ecology and Geobotany, Karl-von-Frisch Strasse 8, 35043, Marburg, Germany

^35^INRAE, ONF, BioForA, 45075 Orléans, France

^36^INRAE, URFM, F-84914, Avignon, France

^37^University of Oulu, Pentti Kaiteran katu 1, 90014, University of Oulu, Finland

^38^Natural Resources Institute Finland, Paavo Havaksentie 3, 90014, University of Oulu, Finland

^39^Institute of Biosciences and BioResources, National Research Council (CNR), via Madonna del Piano 10, 50019, Sesto, Fiorentino, Italy

^40^University of Bordeaux, INRAE, BIOGECO, 33610 Cestas, France

^41^Helix Venture, 33700 Mérignac, France

^42^Université de Lorraine, AgroParisTech, INRAE, SILVA, 54000, Nancy, France

^43^Thünen Institute of Forest Genetics, Sieker Landstr. 2, 22927, Grosshansdorf, Germany

^44^Área de Biodiversidad y Conservación, Universidad Rey Juan Carlos, Calle Tulipán s/n, 28933, Móstoles, Spain

^45^Division of Forestry and Forest Resources, Norwegian Institute of Bioeconomy Research (NIBIO), P.O. Box 115, 1431, Ås, Norway

^46^Centro de Investigación y Tecnología Agroalimentaria de Aragón -Dpto. de Sistemas Agrarios, Forestales y Medio Ambiente (CITA), Avda. Montañana 930, 50059, Zaragoza, Spain

^47^Norwegian Institute of Bioeconomy Research (NIBIO), 8027 Bodø, Norway

^48^Plant Ecology and Geobotany, Philipps-Universität Marburg, 35043 Marburg, Germany

^49^Institute of Applied Genomics (IGA), 33100 Udine, Italy

^50^IGA Technology Services S.r.l., 33100 Udine, Italy

^51^Thünen Institute of Forest Genetics, Eberswalder Chaussee 3a, 15377, Waldsieversdorf, Germany

^52^Austrian Research Centre for Forests (BFW), Seckendorff-Gudent-Weg 8, 1131, Wien, Austria

^53^Institute of Plant and Animal Ecology, Ural branch of RAS, 8 Marta St. 202, 620144, Ekaterinburg, Russia

^54^Institute of Biosciences and BioResources, National Research Council (CNR), via Madonna del Piano 10, 50019, Sesto Fiorentino, Italy

^55^INRAE, ONF, BioForA, F-45075 Orléans, France

^56^Slovenian Forestry Institute, 1000 Ljubljana, Slovenia

^57^Skogforsk, Tomterna 1, 91821, Sävar, Sweden

## Supplementary Material

msad228_Supplementary_Data

## Data Availability

The genetic data underlying this article are available as VCF files at: https://entrepot.recherche.data.gouv.fr/dataset.xhtml?persistentId=doi:10.57745/DV2X0M. Full documentation of bioinformatics pipelines used to generate the VCF files, including SNP filtering steps, are available at https://github.com/GenTree-h2020-eu/GenTree. Code for all other analysis and bioinformatic steps is available at https://github.com/j-e-james/TreeDFEScripts.
