# Supplementary material for: Between but Not Within-Species Variation in the Distribution of Fitness Effects
Source: Mol Biol Evol. 2023 Oct 13;40(11):msad228. doi: 10.1093/molbev/msad228 (PMC10630145; doi:10.1093/molbev/msad228)
Supplement: msad228_Supplementary_Data [file msad228_supplementary_data.zip › SupplementaryFigures_Revised.pdf]

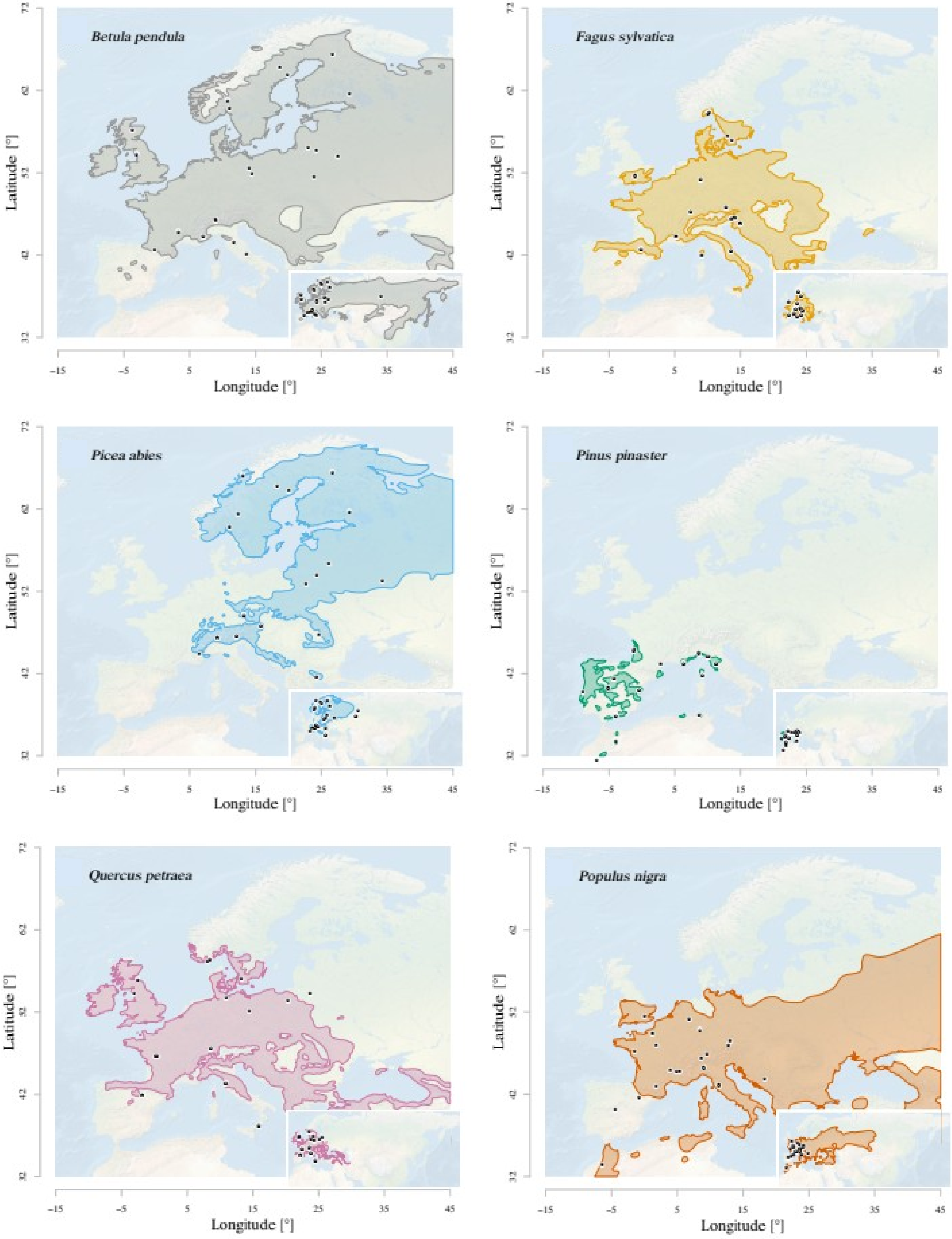

Supple [illegible]
ranges. Species ranges are based on the EUROFORGEN database (www. euroforgen.org).

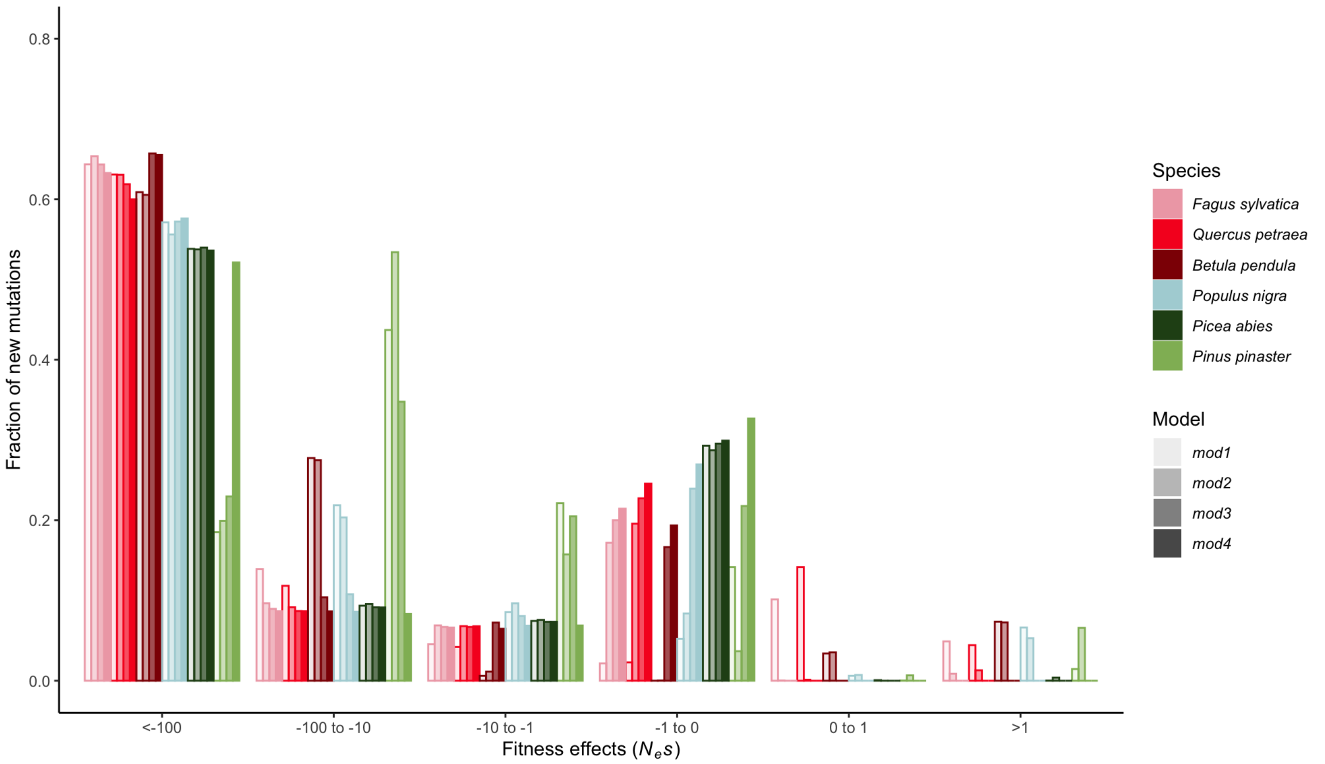

Supplementary figure 2) The discretised DFE (i.e., the fraction of new mutations in each scaled fitness effect ($N_e s$) category), as calculated per model, for each species. The models are as follows: mod1: full DFE, including beneficial mutations, and estimating the rate of error in the inference of the ancestral state (ε_{anc}); mod2: full DFE, including beneficial mutations, without estimating ε_{anc}; mod3: the deleterious-only DFE, including an estimate of ε_{anc}; mod4: the deleterious only DFE without estimating ε_{anc}.

Supplementary text

The scale parameter of the DFE, S_d, is inferred based on fitting a distribution to observed variants, which are dominated by less deleterious and neutral mutations, while mutations that are very strongly deleterious will be rapidly removed by selection and unlikely to be segregating in samples. We conducted simulations to illustrate how changes in S_d affect the SFS of neutral and deleterious mutations, and the distribution of selection coefficients of observed variants segregating within samples.

We ran simulations using SLiM v. 4.0.1 (Haller and Messer, 2023), simulating populations of 500 diploid individuals. Genomes were 100kb long, with a uniform scaled recombination rate of 5e-6, and a mutation rate of 1e-7 per base per gamete. There were two categories of mutations in these simulations; neutral, and deleterious, with neutral mutations constituting one-third of mutations and deleterious mutations constituting the remaining two-thirds of mutations, as is approximately the case for synonymous and non-synonymous sites in coding DNA. The fitness effects of deleterious mutations were drawn from a gamma distribution of shape 0.2 and scale parameter ($N_e s$) of either -1000, -10,000, -100,000 or -1,000,000. Each simulation run was for 10,000 generations, with a sample of 500 genomes taken at the end. Simulations were repeated 50 times per scale parameter value. We then extracted the selection coefficients of all observed segregating mutations in our simulations, and calculated the average neutral and deleterious SFS over all 50 runs. This was achieved using custom python and R scripts available at https://github.com/j-e-james/TreeDFEScripts.

We found (Supplementary Fig. 3) that higher values of S_d reduced population diversity at deleterious sites, particularly at higher frequency categories of the deleterious SFS, and also increased the mean, albeit not the median, *s* of segregating deleterious variants in our simulated population samples, such that a 10-fold increase in S_d resulted in a corresponding increase in mean *s* of approximately 10-fold. However, the mean selection coefficient (*s*) of segregating deleterious variants observed in our simulated population samples was lower than one might expect given the value of S_d, and there was little effect of S_d on the median value of *s*.

References

Haller BC, Messer PW. SLiM 4: Multispecies Eco-Evolutionary Modeling. Am Nat. 2023 May;201(5):E127-E139. doi: 10.1086/723601. Epub 2023 Mar 21. PMID: 37130229.

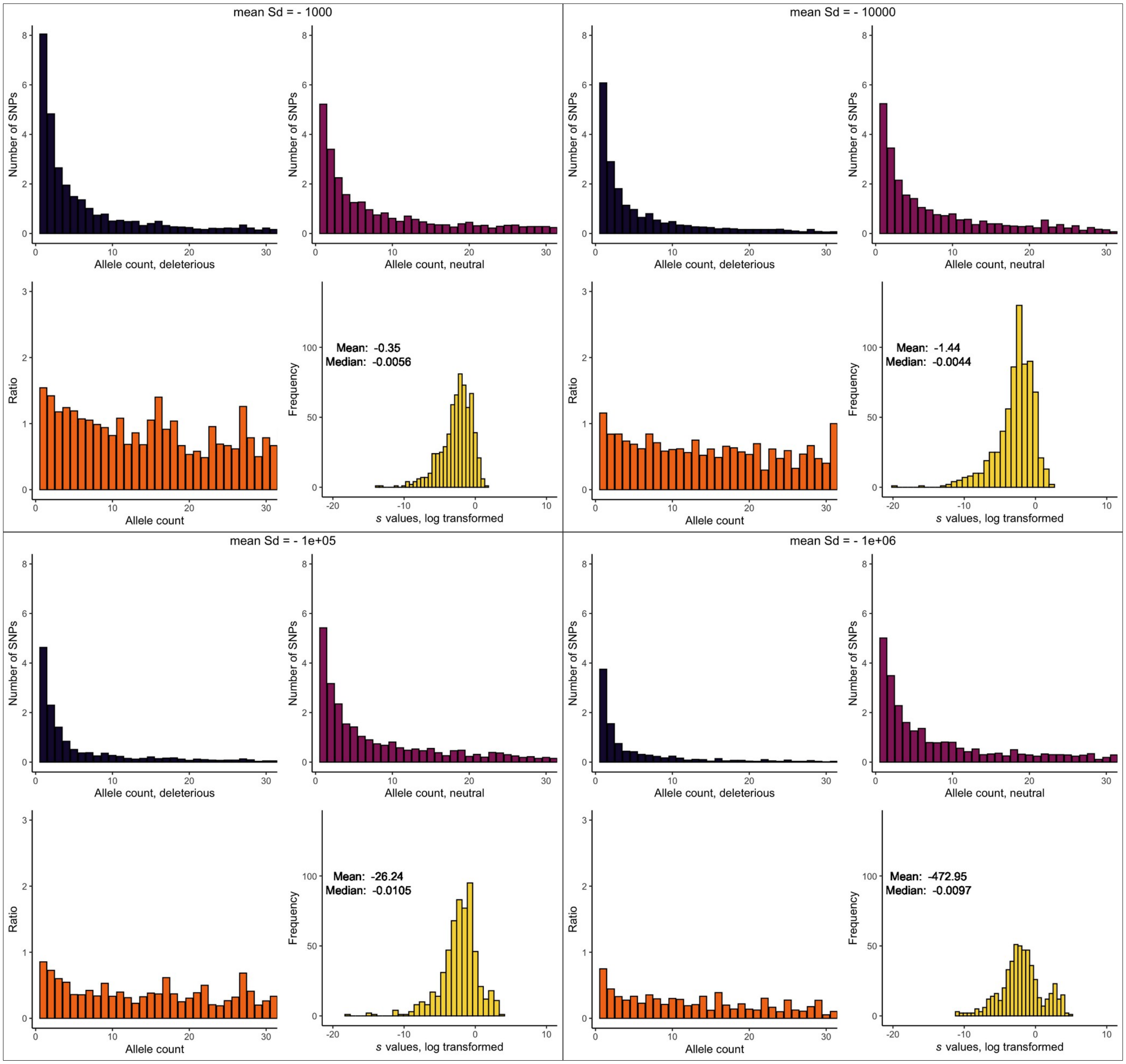

Supplementary figure 3) Simulation results of 500 diploid individuals showing the effect of varying the scale parameter (S_d, the mean selection coefficient of new mutations, scaled by N_e) of the deleterious DFE. For each tested value of S_d, as shown in panel titles, we show the SFS of deleterious (black) and neutral (purple) mutations, averaged over 50 runs, the ratio of deleterious to neutral mutations for each frequency category of the SFS (orange), with plots truncated at 30 on the x axis for ease of visual comparison, and a histogram of the unscaled selection coefficients (s) of the segregating mutations from all 50 runs (yellow). The plot area is annotated with the mean and median s values for the unscaled selection coefficients of segregating mutations in the simulations.

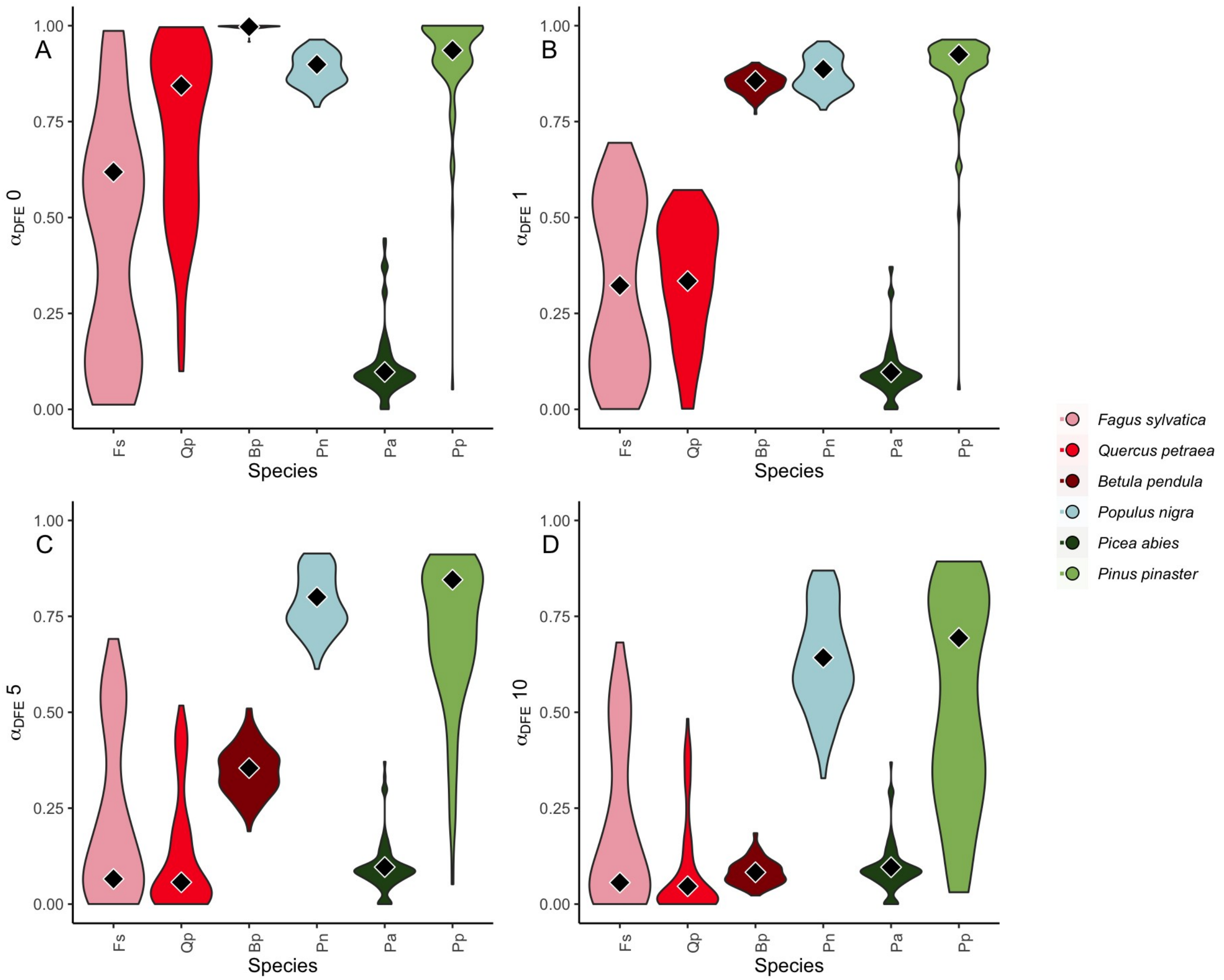

Supplementary figure 4) α_{DFE}, the proportion of substitutions that are expected to be adaptive, plotted per species. The minimum scaled selection coefficient for mutations to be included in the calculation of α_{DFE} varies between plots as follows: A) no lower limit, B) 1, C) 5, D) 10. Black diamonds are the inferred model-averaged parameters, while violins show the 95% confidence intervals, as estimated from model-averaged bootstrap replicates. Plot C corresponds to main figure 3D.

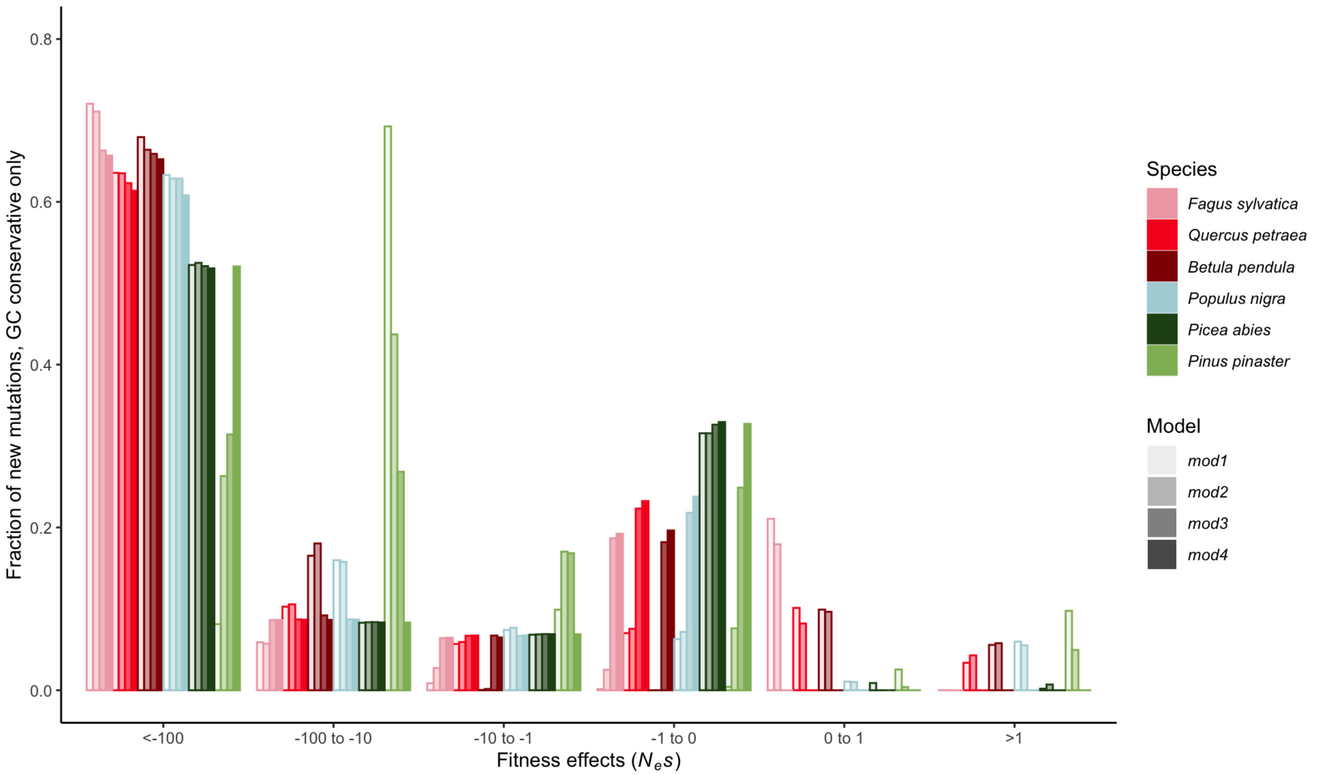

Supplementary figure 5) The discretised DFE (i.e., the fraction of new mutations in each scaled fitness effect ($N_e s$) category), as calculated per model, for each species, for GC-conservative mutations only. The models are as follows: mod1: full DFE, including beneficial mutations, and estimating the rate of error in the inference of the ancestral state (ε_{anc}); mod2: full DFE, including beneficial mutations, without estimating ε_{anc}; mod3: the deleterious-only DFE, including an estimate of ε_{anc}; mod4: the deleterious only DFE without estimating ε_{anc}.

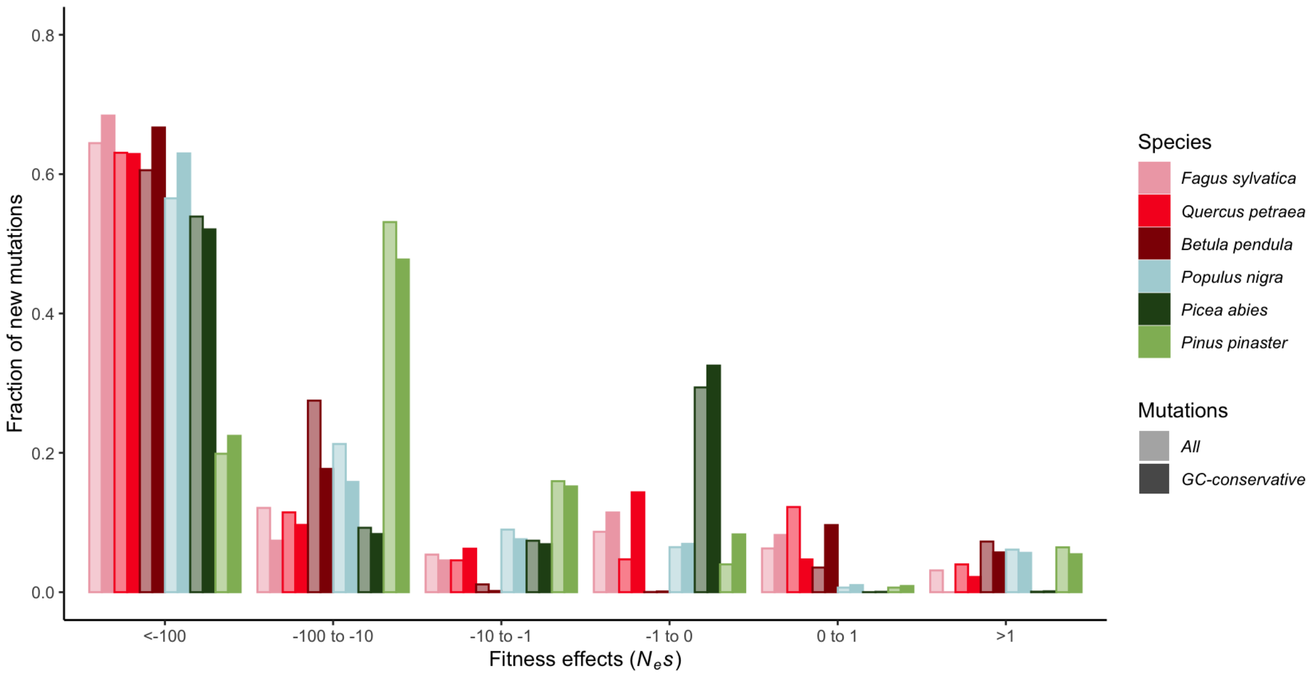

Supplementary figure 6) The model-averaged discretised DFE, i.e., the fraction of new mutations in each scaled fitness effect ($N_e s$) category, as inferred using all mutations or using GC-conservative mutations only.

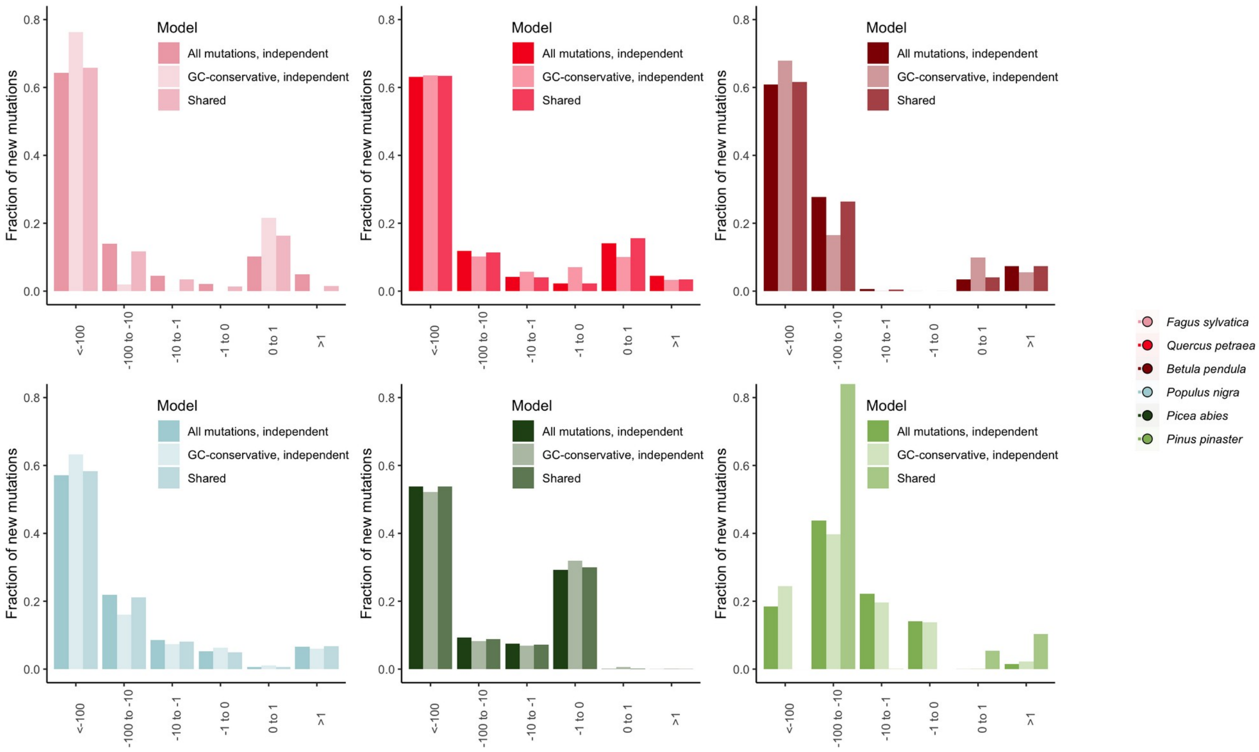

Supplementary figure 7). Discretised DFEs for each species, showing model comparisons for different categories of sites. Darkest bars show the independent fit for all mutations, lightest bars show the independent fit for GC-conservative mutations, intermediate bar shows the fit if the parameters are inferred to be shared across all mutations. We show model fits for the full DFE, including beneficial mutations and an estimate of the rate of ancestral allele misidentification ε_{anc}, for all species.

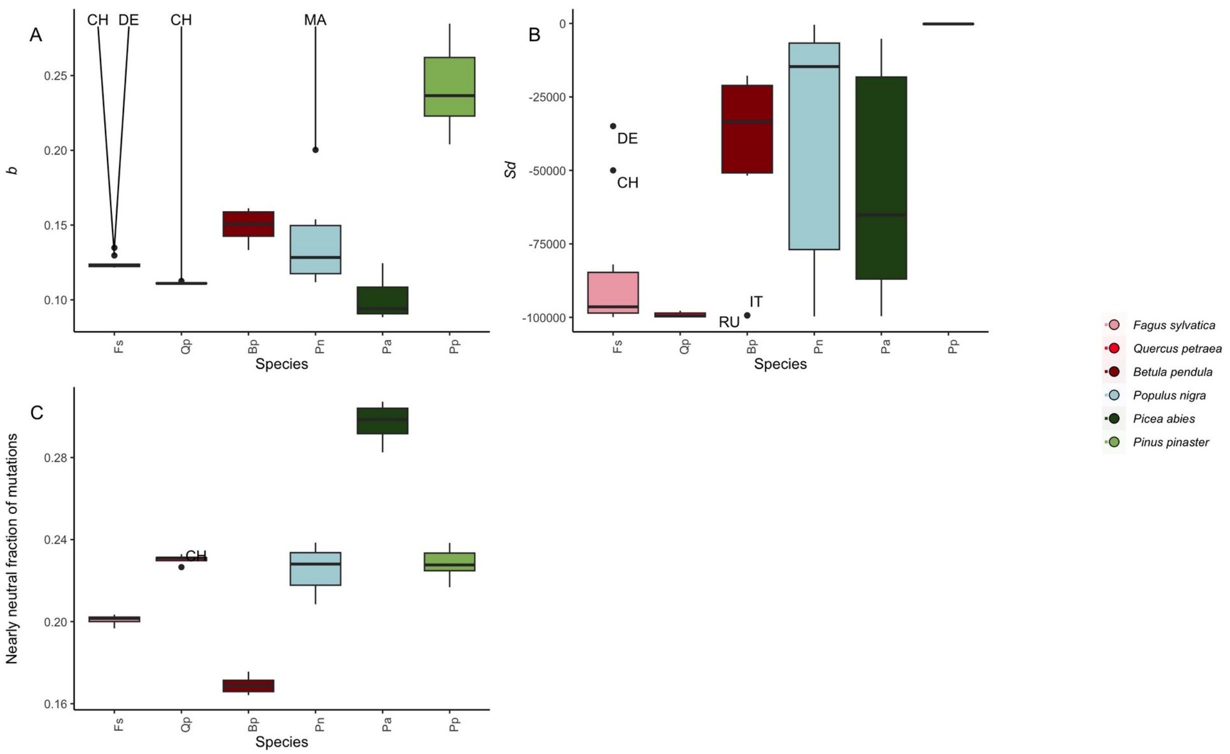

Supplemetary Figure 8). DFE parameters are consistent across populations within a species for the deleterious-only DFE. Shown are the model averaged inferred parameters, for models in which we do not account for the presence of beneficial mutations. We plot the shape (A) and scale (B) parameter of the gamma deleterious distribution of fitness effects, and the proportion of mutations inferred to be effectively neutral (C), i.e. the fraction of mutations for which $-1 < N_e s < 0$. Boxplots show the distribution of values per species, with outlier points indicated as black dots, and labelled by their population codes.

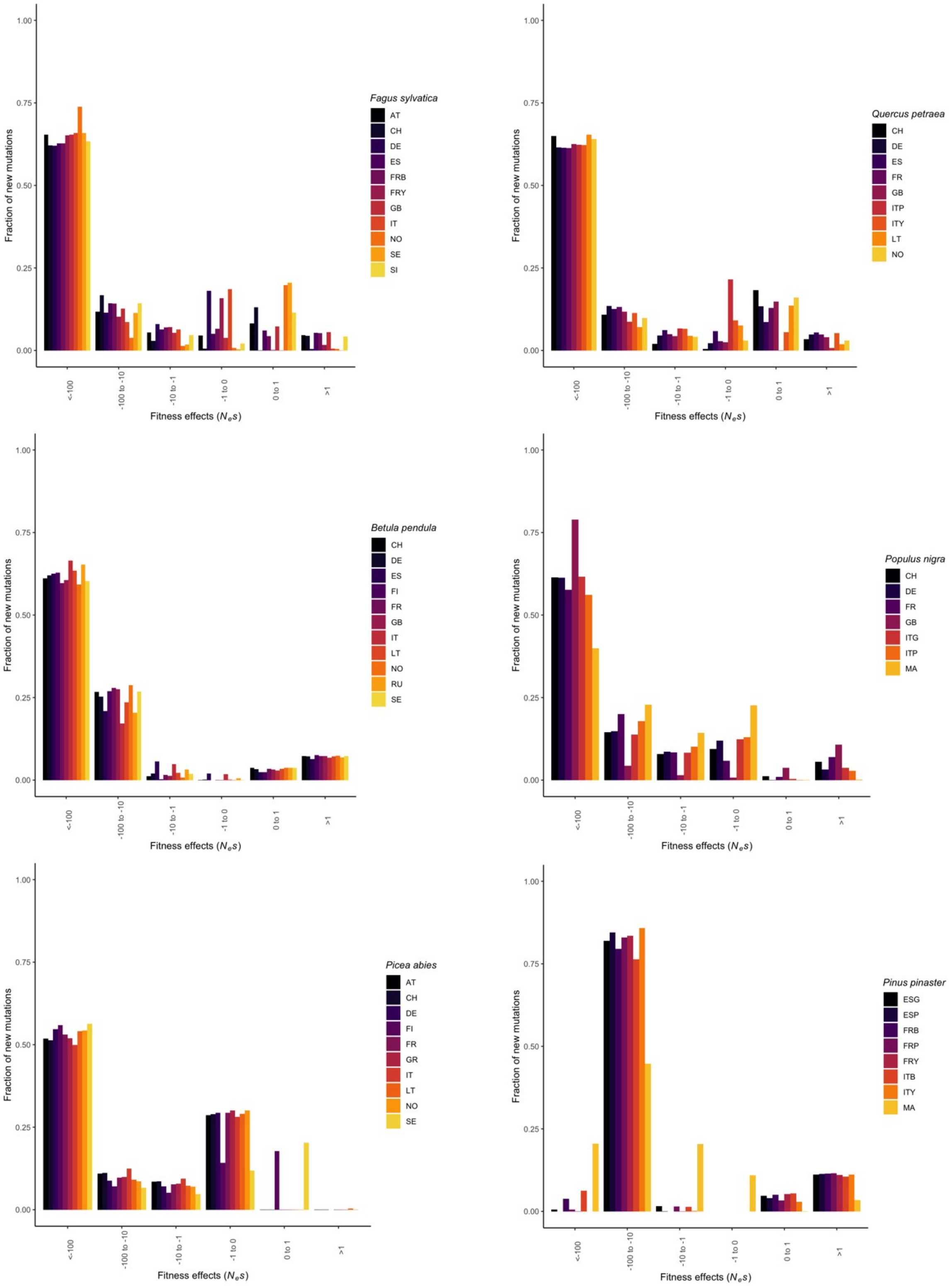

Supplementary figure 9) Discretised DFEs of all populations within species, illustrating differences between populations. Each panel represents a species. Per panel, populations are ordered alphabetically by their letter codes, which always start with the 2-letter country code of their sampling location, as shown in legend. In all panels, we show model fits for the full DFE, including beneficial mutations and an estimate of the rate of ancestral allele misidentification ε_{anc}.

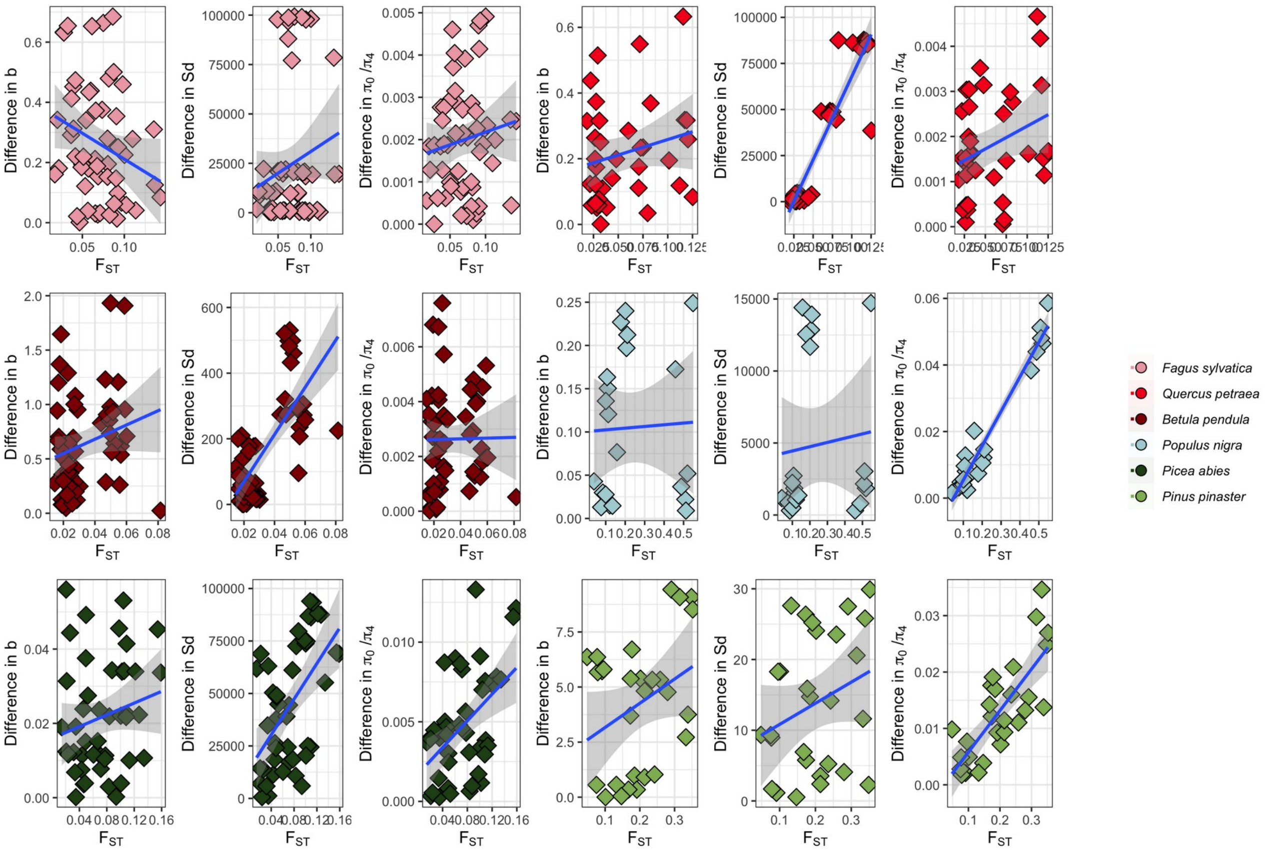

Supplementary figure 10) All pairwise population comparisons per species for the parameters of the gamma distributed deleterious DFE (the shape parameter *b* and the scale parameter S_d) and 0-4 fold synonymous diversity, plotted against Fst. Point colour indicates species, which are consistent with main text figures. Blue lines are linear regression slopes, with grey shaded areas indicating the confidence intervals on the slopes.
